# A systematic review of the relationship between internet use, self-harm and suicidal behaviour in young people: The good, the bad and the unknown

**DOI:** 10.1371/journal.pone.0181722

**Published:** 2017-08-16

**Authors:** Amanda Marchant, Keith Hawton, Ann Stewart, Paul Montgomery, Vinod Singaravelu, Keith Lloyd, Nicola Purdy, Kate Daine, Ann John

**Affiliations:** 1 Medical School, Swansea University, Swansea, Wales, United Kingdom; 2 Centre for Suicide Research, University of Oxford, Oxford, United Kingdom; 3 Oxford Central Child and Adolescent Mental Health Services, Oxford Health NHS Foundation Trust, Oxford, United Kingdom; 4 Centre for Evidence Based Intervention, University of Oxford, Oxford, United Kingdom; 5 Oxford Health NHS Foundation Trust, Oxford, United Kingdom; University of Texas at San Antonio, UNITED STATES

## Abstract

**Background:**

Research exploring internet use and self-harm is rapidly expanding amidst concerns regarding influences of on-line activities on self-harm and suicide, especially in young people. We aimed to systematically review evidence regarding the potential influence of the internet on self-harm/suicidal behaviour in young people.

**Methods:**

We conducted a systematic review based on an electronic search for articles published between 01/01/2011 and 26/01/2015 across databases including Medline, Cochrane and PsychInfo. Articles were included if: the study examined internet use by individuals who engaged in self-harm/ suicidal behaviour, or internet use clearly related to self-harm content; reported primary empirical data; participants were aged under 25 years. New studies were combined with those identified in a previous review and subject to data extraction, quality rating and narrative synthesis.

**Results:**

Forty-six independent studies (51 articles) of varying quality were included. Perceived influences were: positive for 11 studies (38191 participants); negative for 18 studies (119524 participants); and mixed for 17 studies (35235 participants). In contrast to previous reviews on this topic studies focused on a wide range of internet mediums: general internet use; internet addiction; online intervention/treatment; social media; dedicated self-harm websites; forums; video/image sharing and blogs. A relationship between internet use and self-harm/suicidal behaviour was particularly associated with internet addiction, high levels of internet use, and websites with self-harm or suicide content. While there are negative aspects of internet use the potential for isolation reduction, outreach and as a source of help and therapy were also identified.

**Conclusions:**

There is significant potential for harm from online behaviour (normalisation, triggering, competition, contagion) but also the potential to exploit its benefits (crisis support, reduction of social isolation, delivery of therapy, outreach). Young people appear to be increasingly using social media to communicate distress, particularly to peers. The focus should now be on how specific mediums’ (social media, video/image sharing) might be used in therapy and recovery. Clinicians working with young people who self-harm or have mental health issues should engage in discussion about internet use. This should be a standard item during assessment.

A protocol for this review was registered with the PROSPERO systematic review protocol registry: (http://www.crd.york.ac.uk/prospero/display_record.asp?ID=CRD42015019518).

## Introduction

Internet use has a mixed effect on children and young people’s (CYP) well-being, with evidence of increased self-esteem and perceived social support alongside harmful effects such as increased exposure to graphic content and cyber-bullying [1]. High profile cases of cyber-bullying and suicide over the past decade [2] and reports of suicide clusters facilitated by social media [3] have resulted in researchers increasingly focusing efforts on understanding the relationship between internet use and both self-harm and suicide. An early review examining the use of dedicated self-harm support sites in young people found high levels of support available online alongside normalisation of self-harm behaviour [4]. A more recent systematic review relating to self-harm and internet use in young people additionally found evidence of information sharing of methods of self-harm and concealment, a reduced sense of isolation and reinforcement of positive behaviours such as help seeking [5]. The authors concluded that the internet exerts both positive and negative influences on self-harm and provides an opportunity for intervention, which is supported by several more recent studies [6, 7].

While research appears to be beginning to answer some questions regarding the role of the internet for young people who self-harm a number of questions remain unanswered. Authors of the previous review highlighted that research to the date of their review (26^th^ December 2011) described internet use only in relation to use of forums or general use [5]. This is an important limitation since specific internet pathways may represent increased risk. Internet addiction and pro-suicide websites have been suggested as high risk factors facilitating suicidal behaviours, particularly in isolated and susceptible individuals [8]. Additionally, a recent study examining changes in online suicide-related content showed that the results of searches for self-harm have changed over time, with an increasing presence of graphic imagery [6]. The role of such images has been examined in relation to self-harming behaviours [9], as well as relevant content of videos on a popular sharing website [10]. A recent study has also showed that the nature of sites returned varied according to the suicide/self-harm-related search terms use and almost half contain video content. Negative influences such as providing information on methods, encouraging self-harm behaviours and images considered evocative by researchers were common. However, more positive influences including advice on how to seek help was given on over half of identified sites [7]. Further research has highlighted the positive and negative influences of the internet, the potential benefit of directing individuals to healthier online behaviour and the discrepancy in perspectives regarding online activities between adolescent culture and that of many mental health professionals [11].

The previous review also identified a possible relationship between study design and perceived outcome. Qualitative and mixed methods studies tended to report a positive influence of internet use and quantitative designs tended to find a negative impact. Therefore the design and quality of individual studies may have had an impact on perceived outcomes. Following their systematic review [5] the authors highlighted the need for more rigorous research to clarify the positive and negative influences and to focus on the mediating and moderating factors in order to optimise the benefits whilst minimising the potential harm of internet use in young people who self-harm.

The primary aim of this study was to systematically review all research relating to the potential influence of the internet on self-harm/suicidal behaviour in young people, with a particular focus on identifying the factors which determine whether the internet is perceived as positive or negative in its potential influence.

## Method

### Search strategy and selection criteria

This study was a systematic review. The previous electronic literature search, conducted by some of the authors, was up to 26^th^ December 2011 [5]. For the present review, an electronic literature search was conducted (AM) for all articles published between 1^st^ January 2011 and 26^th^ January 2015. A range of databases were searched including: CINAHL; Cochrane Library; EMBASE (excluding Medline journals); HMIC; Medline; NICE; Prospero; PsycINFO; PubMed; SCOPUS. Additional searches were conducted in health improvement sources, topic specific websites (American Association of Suicidology, British Psychological Society, CEBMH, Centre for Mental Health, DH, DHSPSS-NI, MFH, NHS Scotland, Royal College of Psychiatrists, Welsh Government) and meta-search engines (Google/ Google scholar) for these dates only.

The search terms employed in the original review (which included ‘Self harm’, ‘Suicid*, ‘Internet’, ‘Children’, ‘Young People’) were updated to account for the rapidly changing nature of the internet and memes (an image, piece of text, idea that spreads rapidly) in young people. The full details of search terms and sources searched are included in S1 Table. Reference lists of all review articles were manually screened for potential eligible papers. The research team included a number of experts in the field who reviewed searches for any potentially unidentified citations. Experts in the field were also contacted to assist in identification of literature. These included those known to members of the team and others identified from existing literature. They were contacted by email regarding any additional studies and, where relevant, data or full text copies of articles were also requested by email.

Articles were included if they examined internet use by individuals who experienced suicidal ideation, self-harm, or internet use which was clearly related to self-harm content. Any type of online media or activity was considered for inclusion. We included articles that presented primary empirical data and were published in journals. Participants had either to all be aged less than 25 years or have a mean age of 25 years or less. If ages were not stated, participants had to be described as children, adolescents or young adults. Where articles examined more than one age group, only data for the age group fitting these criteria were analysed. Results were not restricted on the basis of location; however, only English language papers were included.

In order to be consistent with the previous review [5] review articles, news articles, single case studies, editorials, comments, conference abstracts and other grey literature, while searched for (e.g. CINAHL, HMIC), were not included in the final analysis. While the inclusion of grey literature can reduce the impact of publication bias, it may also introduce its own set of biases. These include the absence of peer review and the potential that the availability of data would impact on overall results [12]. Included articles from the previous review on this topic 1991–2011 [5] were added to the newly identified eligible articles to provide a complete overview of available evidence. Studies relating to cyber-bullying and self-harm from both the new and existing literature were excluded and will be reviewed in a separate report. This decision was taken due to the number of known quantitative studies related to this specific topic and because it would allow for a more thorough discussion of the literature with the potential for meta-analysis (protocol available from http://www.crd.york.ac.uk/prospero/display_record.asp?src=trip&ID=CRD42017056487).

Two independent reviewers (AJ, AM) manually screened titles. Any disagreements were resolved by consensus. Titles that clearly had no relevance, book chapters, case reports, conference abstracts, comments, editorial, journal notes, grey literature and news sources were excluded at title screen, although reference lists were manually screened for relevant studies. A record was kept of all discarded articles, including the reason for exclusion. Duplicates were removed. The remaining titles with abstracts were then screened for eligibility by the same two researchers. Full text articles were obtained where suitability could not be determined based on the title and abstract. Two researchers independently reviewed the remaining citations (AJ, AM). Any disagreements that could not be resolved through consensus were discussed with a third expert reviewer (KH).

A protocol for this review was registered with the PROSPERO systematic review protocol registry (http://www.crd.york.ac.uk/prospero/display_record.asp?ID=CRD42015019518)

### Data analysis

The data extraction sheet from the previous review [5] was adapted and used to record specific findings from both newly identified articles and those from the previous review (S2 Table). Additional fields were added to account for the greater level of detail in more recent research articles and to allow comparison of internet medium, outcome measured and study design. Studies were divided between four reviewers (AM, NP, AS, VS) and pairs of reviewers’ independently extracted data for each study. Any inconsistencies in data extraction and quality scores were clarified by consensus with at least two study authors. Articles were amalgamated and grouped according to internet medium studied and perceived influence. Positive influences were defined as results indicating perceived reduction of psychological distress, reduced suicidal ideation and self-harm, advice on how to seek help and encouragement to do so. Negative influences were defined as results indicating: increased psychological distress, self-harm or suicidal ideation; information on methods of self-harm/suicide was provided; self-harm behaviours were encouraged. Mixed influences were recorded where a report included both positive and negative influences. Internet media were grouped according to their stated description within articles. These media categories were inductively generated following initial reading and data extraction of papers and were cross checked by two members of the study team (AM and AJ).

Quality of included articles was assessed according to the Critical Appraisal Skills Programme (CASP) [13] as performed previously [5]. This tool assesses various aspects of study design including the study sampled, data collection methods, study design and the clarity and appropriateness of results and conclusions. It also includes items related to potential sources of bias such as from the study population or design.

Due to the range of research questions, methods used, populations and outcomes studied there was a high level of clinical and methodological heterogeneity across studies, precluding any meaningful combination of study results through meta-analysis. Therefore, a narrative synthesis was employed. Based on published guidance [14] this narrative synthesis examined a number of key aspects. Comparisons across studies were made regarding the way in which the relationship between self-harm/suicide and internet use had been identified and analysed; relationships between study results were examined and compared across the studies; the influence of heterogeneity was further explored including theoretical variables, differences in baseline characteristics of populations, measures employed and outcomes studied.

## Results

**Fig 1:**
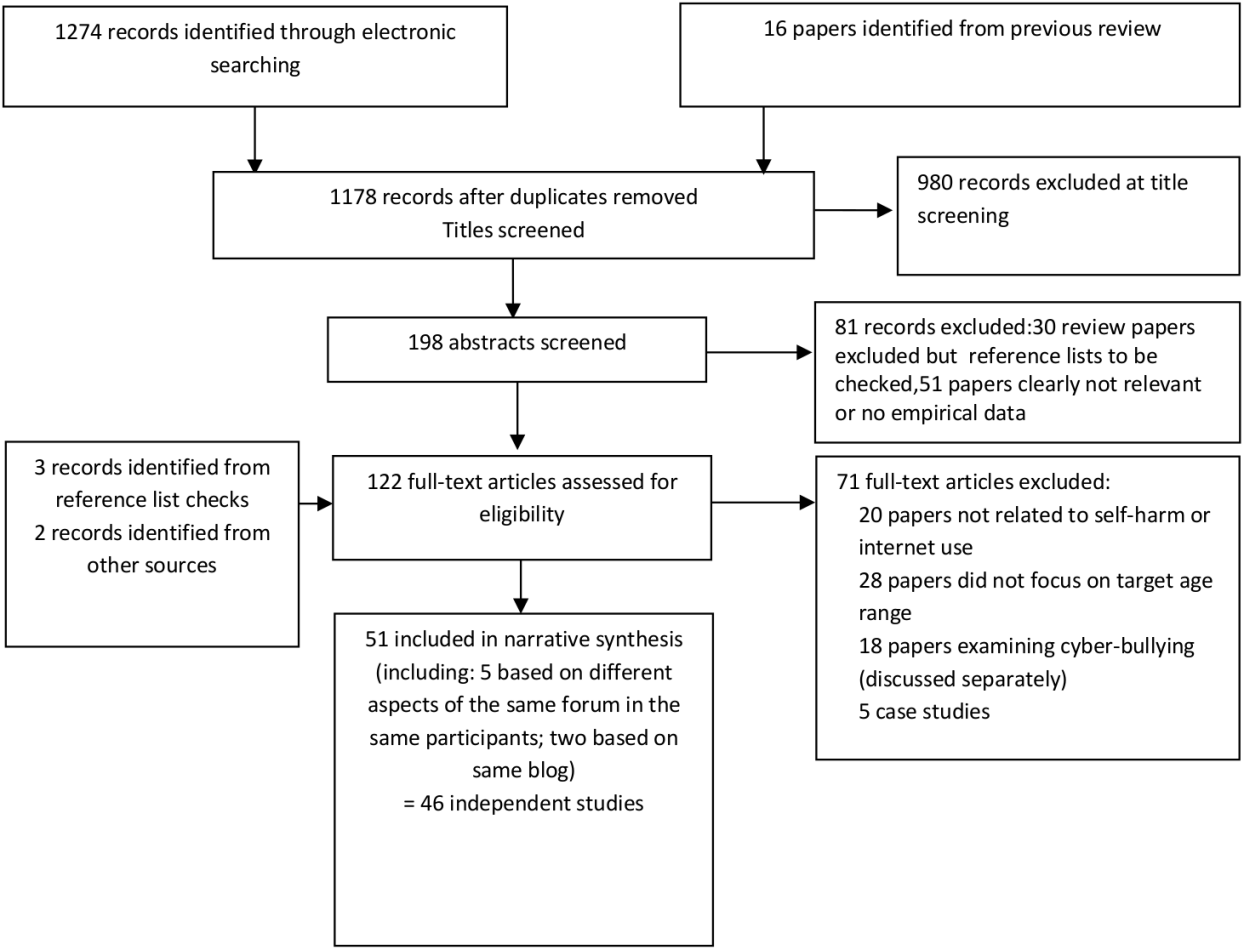
Flow of information through the evidence review.

Fig 1 shows the results of the search strategy and screening process. A total of 51 articles (from 46 independent studies) were included in the review. A summary of included articles by internet medium and perceived influence can be seen in Table 1. Studies were based in the USA (n = 10), UK (5 studies, 9 articles), Canada (n = 5), Japan (n = 3), Korea (n = 3), Australia (n = 2), New Zealand (n = 2), Sweden (n = 2), China (n = 1), Germany (n = 1), Israel (n = 1), Northern Ireland (n = 1), South Africa (n = 1), Taiwan(n = 1) and Turkey (n = 1), with the remaining 7 studies (8 articles) from multiple countries. A total of 192,950 individuals participated. Forty-four of the 51 articles had more than 50% female participants. Eleven studies [15–25] examined content of forum posts or websites in which participants were described only in terms of demographics of site users. One study [26] examined rates of suicide by age group with no further description of the number of participants. Using the CASP quality score 17 articles were assessed as high quality, 19 as medium quality and 15 as low (S3 Table). The quality of articles varied by study design with a greater proportion of quantitative (13/21) than qualitative ones (0/18) rated as high quality. Number of studies, number of articles, study design and quality varied across internet media (Table 2).

**Table 1:** Summary of included studies.

Internet medium	Lead Author, year, country	Population (N, %female)	Aims, Objectives	Results, outcome	Outcome	Design,Quality score
**General use**	Carew, 2014 [27], Canada and USA	Internet users (28805; 64)	To investigate mental health information seeking online, and to identify differences within age groups and geographical location	A 200% increase in online activity regarding mental health was identified (between 2006 [baseline] and 2010). Adolescents were most likely to initiate conversation about depression followed by anxiety, alcohol, suicide, sexting and marijuana. Adolescents tended to discuss concerns through the use of personal stories.	Positive	Quantitative High
	Casiano, 2012 [28],Canada	Canadian young people aged 12–19 (9137; 49)	To examine to association between quantity of media use and health outcomes in adolescents	No significant association between any form of media and suicide ideation (internet use OR 0.98, 95% CI 0.83–1.16)	Positive	QuantitativeLow
	Carli, 2014 [29], 11 European countries	School based adolescents in eleven European countries (12395; 55)	To explore the prevalence of risk behaviours (excessive alcohol, drug use, truancy etc.) and their association with psychopathology and self-destructive behaviours	Latent class analysis identified three groups of adolescents: high risk, including pupils who scored high on all risk behaviours; low risk including pupils with low frequency of behaviours and invisible risk. This 'invisible risk' group was found to score high on use of media and have similar prevalence of suicidal thoughts/psychopathology as ‘visible risk’ group. The invisible risk group were at significantly higher risk than the low risk group for non-suicidal self-injury (Relative risk ratio (RRR) = 1.40; 95% CI 1.13 1.84), suicidal ideation (RRR = 1.29; 95% CI 1.12–1.48) and suicide attempt (RRR = 1.22; 95% CI 1.22–2.35).	Negative	QuantitativeHigh
	Hagihara, 2012 [26], Japan	Young adults in Japan; Rate of suicide;	To examine the association between suicide-related searches and the incidence of suicide on young adults in Japan	Association between Internet suicide-related searches and the incidence of suicide in Japan (over 77 months): the terms "hydrogen sulphide", "hydrogen sulphide suicide", and "suicide hydrogen sulphide suicide" at (*t*-11) were related to the incidence of suicide among people aged in their 20s (*P* = 0.005, 0.005, and 0.006, respectively).	Negative	Quantitative Low
	Katsumata, 2008[30], Japan	Japanese high school students (590;49)	To investigate the association between the experience of using electronic media and suicidal ideation in Japanese adolescents.	Suicidal ideation was significantly associated with anxiety about not getting email replies (OR 2.06; 95% CI1.33–3.20), and searching online for information about suicide and self-harm (OR 5.11; 95% CI 2.43–10.71) and hurtful experiences on the web (OR 1.71; 95% CI 1.03–2.84)	Negative	QuantitativeLow
	Kim, 2012 [31], Korea	Korean middle and high school students (75066; 47)	To consider the association between internet using time for non-educational purposes and adolescent health	Internet non-users (NIU) and heavy internet users (HIU) were found to be high risk groups when compared with moderate internet users (MIU) on multiple mental health measures. Suicide ideation was significantly higher in HIU and NIU (females: HIU = 43.4%; NIU 25.8%; OIU 21.8% (P<0.001); males: HIU 26.4%; NIU 16.7%; OIU 13.6% (P<0.001)) as was rate of attempted suicide (females: HIU 13.9%; NIU 7.3%; OIU 5.2% (P<0.001); males: HIU 10%; NIU 4.9%; OIU 2.4% (P>0∙001))	Negative	Quantitative High
	Mitchell, 2007 [32], USA	American internet users aged 10–17 (1500; 81)	To explore internet use and interpersonal interactions of youth reporting self-harm	Youth who self-harm engaged in more risky online behaviours than those who did not including using chat rooms (57% compared with 29%) and to have a close relationship with someone they met online (38% vs. 10%)	Negative	QuantitativeHigh
	O'Connor, 2014 [33], Northern Ireland	Adolescents in Northern Ireland (3596; 48)	To determine the prevalence of self-harm and associated factors	Self-harm was found to be associated with internet/social media as well as variety of other factors including exposure to the Northern Ireland conflict. In total 15% of girls and 26% of boys endorsed either the internet or social networking sites as factors that influenced their self-harm.	Negative	Quantitative High
	Robertson, 2012 [3], New Zealand	New Zealand adolescents (8; 88)	To describe an adolescent suicide cluster and the possible role of online social networking and text messaging as sources or contagion and obstacles to recognition of a potential cluster	These cases did not belong to a single school but were linked by social networking sites including memorial pages. This facilitated the rapid spread of information and made recognition and management of a possible cluster more difficult	Negative	Qualitative Medium
	Collings, 2011 [34], New Zealand	New Zealand adolescents (71; 79)	To describe the influences of media on suicidal behaviours, from the perspectives of young people.	Participants considered some interactive media supportive. 80% (n = 12) of those who used violent methods of self-harm had been exposed to suicide content via the internet before the incident	Both Positive and negative	Mixed MethodsHigh
	Duggan, 2012 [20], Canada	Scope and nature of self-harm content across various internet mediums	To examine the scope and nature of self-harm content across informational/interactive websites, social networking websites and YouTube	Results suggest that peer driven websites are accessed more often than professionally driven websites. Self-harm is strongly represented among social networking websites and YouTube evidenced by large group memberships and video counts. The search terms yielded 41 dedicated groups on Facebook with memberships ranging from 2 to 4,686. The same search yielded 206 groups on MySpace with group membership ranging from 2–1653. Searches on YouTube produced 2,290 videos. Characteristics of groups, videos and posters are described.	Both positive and negative	QuantitativeMedium
	Dunlop, 2011 [35], USA	Young people aged 14–24 (719)	To determine whether online news and social networking sites, expose young people to suicide stories that might increase suicide ideation	Online sources of information were quite common (reported by 59% of participants). Social networking sites were frequently cited as sources but were not linked to increases in ideation. However online discussion forums were associated with increases in suicide ideation	Both positive and negative	QualitativeLow/medium
**Internet addiction**	Kaess, 2014 [36],11 European countries	School based adolescents in eleven European countries (11356; 57)	To investigate the association between pathological internet use, psychopathology and self-destructive behaviours	Suicidal behaviours, depression, anxiety, conduct problems and hyperactivity/inattention were significant and independent predictors of pathological internet use (Suicidal ideation coefficient 0.324, 95% CI 0.251–0.397, P <0.001; Suicide attempts coefficient 0.552, 95% CI 0.207–0.896, P = 0.001).This association is significantly influenced by country and gender.	Negative	QuantitativeHigh
	Kim, 2006 [37], Korea	High school students in Korea (1573; 65)	To elucidate the relationship between internet addiction, depression, and suicidal ideation	Internet addiction scores were positively correlated with suicidal ideation in non-internet addicts, possible addicts and internet addicts (non-addicted r = 0.111, p = 0.001; possible addicted r = 0.147, p < 0.001; internet addicted r = 0.448, p<0.001)	Negative	QuantitativeHigh
	Lam, 2009 [38], China	Adolescents in china (1639;55)	To examine the association between internet addiction and self-harm	Moderately or severe internet addiction was associated with higher incidences of self-harm (adjusted OR 2.0, 95% CI 1.1–3.7).	Negative	Quantitative High
	Lin, 2014 [39], Taiwan	Taiwanese adolescents aged 12–18 years (9510; 52)	To examine the associations of suicidal ideation and attempt with internet addiction and activities	Internet addiction was significantly associated with suicidal ideation (OR 1.25, 95% CI 1.08–1.44) and suicide attempt (OR 1.59, 95% CI 1.29–1.96). Specific internet activities associated with increased and decreased risk	Negative	QuantitativeHigh
	Park, 2013 [40], Korea	Korean middle and high school students (795; 68)	To evaluate a)associations between problematic internet use and depression, bipolar disorder symptoms and suicidal ideation; and b) whether mood disorders mediate the relationship between suicidal ideation and problematic internet use	Presence of problematic internet use significantly associated with suicidal ideation (OR = 5.82, 95% CI = 3.30–10.26, p<0.001) as well as depression (OR = 5.0, 95% CI = 2.88–8.66, p<0.001) and probably bipolar disorder (OR = 3.05, 95% CI 0.96–9.69, p = 0.059). Problematic internet use was found to predict suicidal ideation (ß = 0.115, 95% CI = 0.052–0.193, p = 0.006). Conversely suicidal ideation was found to predict problematic internet use ((ß = 0.215, 95% CI 0.089–0.346, p = 0.006). Complex transactional relationship.	Negative	QuantitativeMedium/high
	Aktepe, 2013 [41], Turkey	High school students in Isparta (1897; 43)	To measure the prevalence of internet addiction and to detect related socio-demographic factors	The prevalence of possible internet addiction was found to 14%. A significant association between problematic internet use and self-harm was found (ß = 0.574, OR = 1.79, 95% CI 1.30–2.43, P <0.001). Adolescents with possible internet addiction were also found to have low levels of loneliness and high levels of life satisfaction.	Both positive and negative	QuantitativeHigh
	Messias, 2011 [42], USA	Students aged 14–18 years; (16124; N/A)	To investigate the association between excessive video game/internet use and teen suicidality	Teens who reported more than 5 hours a day of video game/internet use had a significantly higher risk of suicidal ideation (OR = 1.7, 95% CI 1.3–2.1) and suicide planning (OR = 1.5, 95% CI 1.1–1.9). Authors find a potential protective influence of low video game use compared with no use.	Both positive and negative	QuantitativeHigh
**Sources of help**	Hetrick, 2014^a^ [43], Australia	Melbourne high school students experience suicidal ideation; (21)	To investigate the usefulness of an internet- based CBT programme	Over the course of the intervention negative problem-solving orientation improved (t = 4.38, p < 0.0005) and students relied less on emotion focused coping strategies. Adolescents rated the problem-solving and cognitive restructuring modules as particularly helpful.	Positive	QuantitativeMedium
	Hetrick, 2015 [44], Australia	Australian young people aged 15–24 (15)	To develop and examine the feasibility of an online monitoring tool of depression symptoms, suicidality and side effects	Results show that an online monitoring tool is potentially useful as a systematic means for monitoring symptoms of depression and suicidality, but further research is needed including how to embed the tool within clinical practice	Positive	Mixed methodsMedium
	Mar, 2014 [45] UK	Individuals age 16–24 who had experienced suicidal ideation (23; 96)	To explore youth consumer preferences for online interventions targeting depression and anxiety	Youth positively received the idea of e-mental health services. Noted preferences for services that are simple to use, interactive and include support through an online community.	Positive	Mixed methodsLow/medium
	Saulsberry, 2013 [46], USA	Adolescents screening positive for depression in primary care (83; 57)	To test an internet program for young people with depression	Participants demonstrated significant within-group decreases in depression and self-harm ideation (any thoughts of self-harm in previous two weeks 14.46% at baseline compared with 4.82% at 1 year follow-up)	Positive	QuantitativeHigh
	Barton, 2013 [47], USA	College students (106; 55)	Study examined responses to open-ended email vignettes from a fictitious friend exhibiting depressed, irritable or suicidal communications	Results indicate student’s preferences for solving fictitious peer problems personally rather than professionally. Patterns of help-giving and sex differences varied by condition	Both positive and negative	QualitativeMedium
	Whitlock, 2013 [48], USA	College students (14372; 43)	To examine the impact of questions regarding self-injury, suicide and psychological distress in a web-based survey on respondents	Less than 3% of individuals reported negative survey experiences. Individuals with relevant personal experience reported greater discomfort with the survey yet were also significantly more likely to report that it caused them to reflect on their lives	Both positive and negative	Mixed methodsHigh
**Social media**	Belfort, 2012 [49], USA	Adolescents presenting to hospital with self-harm (1350; 75)	To describe key similarities and difference among adolescents who communicated their suicidality via electronic Vs. other means	Numbers of electronic communication of suicidality increased over time from 8.3% in 2005 to 55.6% in 2009. Patients who communicated suicidality electronically more likely to do so to a peer (67% compared with 7% of those communicating by other means).	Negative	QuantitativeLow
	Cash, 2013 [24], USA	MySpace users aged 13–24 (64; 40)	To explore the ways in which adolescents use MySpace to comment on their suicidal thoughts and intention	Comments referenced a significant amount of hopelessness, despair and desperation. Adolescents use public web sites to display comments about their suicidal thoughts, behaviours and intentions.	Negative	QualitativeLow/medium
	Zdanow, 2012 [21], South Africa	Analysis of self-harm groups on Facebook	To analyse the representation self-harm on dedicated Facebook groups	Content analysis of two groups revealed glorification and normalisation suicidal behaviours. Potential for social networking sites to be used as a tool for the promotion and encouragement self-harm	Negative	QualitativeLow
	Sueki, 2015 [50], Japan	Young adult twitter users (1000; 61)	To examine the association between suicide-related tweets and suicidal behaviour to identify suicidal young people on the internet	Logistic regression analysis showed that tweeting 'want to die' was significantly associated with history of suicidal ideation (OR = 2.53, 95% CI 1.61–3.99) having a suicide plan (OR = 2.55, 95% CI 1.56–4.17) and attempting suicide (OR = 1.67, 95% CI 0.95–2.94). Tweeting 'want to commit suicide' was significantly related to history of self-harm (OR = 1.87, 95% CI 1.03–3.41), having a suicide plan (OR = 1.92, 95% CI 1.07–3.46) and attempting suicide (OR = 3.48, 95% CI 1.89–6.42). Having a twitter account and tweeting daily were not associated with suicidal behaviour	Both positive and negative	Mixed methodsMedium/High
**Forum**	Baker, 2008 [51], UK	Users of self-harm discussion forums (10, 50)	To explore the accounts of young people who self-harm and use forums	Forums were used positively for support and communication. Some participants report a reduction in the incidence of self-harm	Positive	QualitativeLow
	Barak, 2006 [52], Israel	Users of self-harm discussion forums (20, 75)	To assess whether the degree of forum involvement affected distress levels	Levels of forum involvement was association with lower levels of distress, however levels of distress did not improve over three months (F = 2.10; df = 2, 787)	Positive	Mixed MethodsLow
	Jones^b^, 2011 [53], UK	Users of a self-harm forum built for research (77, 95)	To explore what young people who self-harm think about online self-harm discussion forums	Participants claimed to learn more about mental health issues from online forums than from information sites, find it easier to talk about self-harm to strangers than to family or friends and preferred to talk online than in person.	Positive	Mixed MethodsMedium
	McDermott, 2013 [15], UK	Analysis of forum posts	To use qualitative methodology to examine internet forums where LGBT^c^ youth discuss self-harming	This methodology can address some research dilemmas by generating diverse samples and a different type of unmediated complex data. Online data can enhance understanding of hard-to-reach youth	Positive	Qualitative Low/Medium
	Owens^b^, 2012 [54], UK	Users of a self-harm forum built for research (77; 95)	To bring together young people who self-harm and health professionals online	The young people were keen to share their experiences and supported one another during crises. Health professionals did not actively participate in forums due to reported lack of confidence and concerns relating to workload and duty of care.	Positive	Mixed methodsMedium
	Sharkey^b^, 2012 [55], UK	Users of a self-harm forum built for research (77, 95)	To use discourse analysis and the concept of face-work as a framework to understand interactions in a self-harm support forum	Use of a range of mitigation devices found suggesting that the young people orient a 'protective line' in their supportive interactions. This may enable a more trusting, open context for support.	Positive	QualitativeMedium
	Smithson^b^, 2011 [56], UK	Users of a self-harm forum built for research (77, 95)	To explore how young adults became members and sustained membership in a self-harm support forum	Participants displayed expectations about appropriate ways of discussing self-harm, responses and advice. Participants were active in shaping interaction on the forums requesting input from moderators.	Positive	QualitativeLow
	Smithson^b^, 2011 [57], UK	Users of a self-harm forum built for research (77, 95)	To investigate the nature of problem presentation and responses in an online support forum	Analysis highlighted the tendency to offer advice where it was not asked for and the mundane 'safe' nature of advice	Positive	QualitativeLow
	Whitlock, 2006 [16], USA	Analysis of forum posts	To investigate the prevalence and nature of self-injury forums, to explore the content, role and influence of discussion forums	Informal support was the most common type of exchange (28.3% of posts). Concealment of practice (9.1%), perceived addictiveness (8.9)and formal help-seeking (7.1) were also discussion themes	Positive	Mixed MethodsHigh
	Eichenberg, 2008 [58], Germany	Users of suicide discussion forums (164; 50)	To assess the assumption that suicide message boards are harmful.	Both constructive (e.g. help-seeking) and destructive (e.g. finding a suicide partner) motives were identified. A significant reduction in suicidal thoughts was found following forum use (effect size *d =* 0.72 (t [144] = 9.2; *p* < 0.01). Unable to directly infer cause.	Both positive and negative	QuantitativeHigh
	Franzen, 2011 [17], Sweden	Qualitative study of a Swedish-speaking web community connected to self-harm	To analyse how self-injuring men and women construct themselves as cutters	Two main interdependent discourses are identified within the web community: the 'normalising' and the 'pathologizing'.	Both positive and negative	QualitativeLow
	McDermott, 2015 [18], UK	Analysis of web-based discussions	To utilise qualitative virtual methods to investigate LGBT^c^ youth web-based discussions about seeking help for suicidal feelings and self harming	Young people wanted assistance but found it difficult to ask for help and articulate emotional distress	Both Positive and negative	QualitativeLow/medium
	Sueki, 2012 [59], Germany and Japan	Users of suicide forums in Japan and Germany (301, 54)	To analyse the cross-cultural use of suicide forums in Japan and Germany	Factor analysis demonstrated two motives: mutual help and suicide preparation. Suicidal thoughts did not worsen with forum use and there was no difference in demographics, motives or effects of suicide forums between Germany and Japan	Both Positive and negative	QuantitativeLow/ medium
	Westerlund,2013 [19], Sweden	Analysis of young adult forum posts	To examine conversations about suicide on discussion forums	Most participants communicate based on a need to gain acceptance and understanding. However there was also exchange of suicide methods and encouragement to go ahead with suicide plans	Both positive and negative	QualitativeLow
**Website with suicide/self-harm content**	Lewis, 2011 [25], Canada	Authors and users of self-harm websites (71; 79)	Examination of the content of non-suicidal self-injury web sites	Websites depict self-harm as an effective coping mechanism (92%), addictive (87%) and not always painful (24%). Almost all websites contained melancholic tones (83%) and several contain graphic imagery (30%). Overall it is suggested that such sites may normalize and reinforce self-harm	Negative	QualitativeMedium
	Harris, 2013 [60], Cross cultural (UK Europe, Canada, Australia, New Zealand and others)	Self-selected users of self-harm websites (329; 92)	To explore the reasons people visit self-harm websites or forums; beliefs regarding these sites; how the use of such sites modulates self-harm and other impacts of these sites on the lives of those who use them	65.6% of participants visited sites at least twice a week, 78.2% used sites to find information and 68.4% to participate in forums. Positive effects of website use such as gaining help and support and reduction in self-harm behaviours were reported by a large number of participants. However smaller numbers reported negative effects including worsened self-harm	Both positive and negative	Mixed methodsHigh
**Video/image sharing**	Lewis, 2012 [22], Canada	Analysis of comments on YouTube videos related to self-harm	To examine viewers comments on non-suicidal self-injury YouTube videos and determine potential risks and benefits of such videos	Viewer’s responses to videos may maintain the behaviour with admiration of video quality (21.95%), message (17%) and up-loader (15.40%) common. Comments rarely encourage or mention recovery (<3%). Sharing experiences online is a strong motivator for viewers of self-harm related videos	Negative	QualitativeMedium/High
	Grzanka, 2014 [23], USA	Critical discourse analysis of online videos	To investigate a mass-mediated campaign against a perceived increase in suicides among gay youth in America	Analysis of videos showed a neoliberal frame that places the burden of a 'better' life onto youth who are instructed to endure suffering in the interest of inevitable happiness	Both positive and negative	QualitativeLow
	Lewis, 2011 [10], Canada	Posters of self-harm videos on YouTube (100; 95)	To examine the accessibility and scope of non-suicidal self-injury videos online	The top 100 videos were viewed over 2 million times and most were accessible to a general audience. Viewers rated videos highly (M = 4.61; SD 0.61 out of 5.0) and selected videos as a favourite over 12000 times. Explicit imagery common (64% of videos) with many videos not warning about this content	Both positive and negative	Mixed methodsMedium
	Sternudd, 2012 [61], UK, USA Europe	Young people who self-harm (52; 87)	To examine reasons for, and reactions to producing/viewing self-harm images online	Informants reported effects images was alleviating rather than triggering. When interpreting statements about images 40% were positive and 25% were negative. To publish them was a way of sharing experiences with others and to give or receive help. Participants emphasised that the outcome of viewing these photos varies by individual and situation	Both positive and negative	Mixed methodsLow
**Blogs**	Castro^d^, 2012 [62], Portugal and Brazil	Authors of Portuguese language blogs (11, 82)	Analysis of pro-anorexia blogs to systematize and categorize their characteristics, content and messages	Blogs can have negative consequences as a result of sharing harmful information about fasting, drugs, self-harm and suicide	Negative	QualitativeLow
	Castro^d^, 2013 [63]^,^ Portugal and Brazil	Authors of Portuguese language blogs (11, 82)	Analysis of pro-anorexia blogs to better understand the influence of social and cultural pressures	Positive relationship found between social and cultural pressures and engaging in self-harming/destructive behaviours	Negative	QualitativeLow

a: Part of a three part series related to online interventions. Subsequent two papers while cited in press have publication dates outside of current search

b: Five reports related to the same self-harm forum study (Sharptalk)

c: Lesbian Gay Bisexual and Transgender

d: Two reports based on the same set of eating disorder blogs

**Table 2:** Research methodology and CASP quality score by internet medium.

Variable		General use (12 papers; n = 131887(papers; n^a^)	Internet addiction (7 papers; n = 42894) (papers; n^a^)	Sources of help (6 papers; n = 14620) (papers; n^a^)	Social media (4 papers; n = 2414)(papers; n^a^)	Forums (14 papers^b^; n = 572)(papers; n^a^)	Self-harm website (2 papers; n = 400)(papers; n^a^)	Video/ image sharing (4 papers; n = 152)(papers; n^a^)	Blogs (2^b^ papers; n = 11)(papers; n^a^^c^)	Total (51 papers; 46 studies; n = 192950)(papers; n^a^)
**Methodology**	**Quantitative**	9; 131089	7; 42894	2; 104	1; 1350	2; 465	0; 0	0; 0	0; 0	21; 175902
	**Qualitative**	2; 727	0; 0	1; 106	2; 64	8; 10	1; 71	2; 0	2;11	18; 989
	**Mixed**	1; 71	0; 0	3; 14410	1; 1000	4; 97	1; 329	2; 152	0; 0	12; 16059
**CASP quality score**	**High**	6; 121433	6; 42099	2; 14455	0; 0	2; 164	1; 329	0; 0	0; 0	17; 178480
	**Medium**	3; 727	1; 795	4; 165	2; 1064	6; 378	1; 71	2; 100	0; 0	19; 3300
	**Low**	3; 9727	0; 0	0; 0	2; 1350	6; 30	0; 0	2;52	2;11	15; 11170

a: number of independent participants, i.e. participants contributing to more than one paper are only counted once

b: includes 5 reports related to the same self-harm forum (sharptalk)

c: includes two reports based on the same set of eating disorder blogs

The sampling of participants varied greatly between studies, each introducing potential selection bias. For example, study participants were recruited via dedicated online support forums [51–59, 61]; emergency departments [49]; other healthcare settings [44–46]; through digital metrics [27]; and through large school-based and community surveys [28–31, 33, 36–42].

A number of outcomes were assessed in the studies, including levels of self-harm and suicidal behaviours, mental disorders, internet addiction, levels of loneliness and insomnia, the potential to recruit participants for research [15] and the nature of online information seeking [27] (S4 Table). Measures ranged from study-specific self-report questionnaires or content analysis themes to validated scales to assess suicidal behaviours, internet use, mental disorders and well-being e.g. Internet Addiction Scale, Beck Depression Inventory, Strength and Difficulties Questionnaire, Suicidal Ideation Questionnaire.

Perceived influences were: positive, 15 articles, 11 independent studies, n = 38,191 participants; negative, 19 articles, 18 independent studies, n = 119,524 participants; mixed, 17 articles, 17 independent studies, n = 35,235 participants. Table 3 summaries the mechanism of perceived influence by internet medium.

**Table 3:** Summary of mechanisms of perceived influence by internet medium.

	**Influence**			**Mechanism**	
**Medium**	Positive (reports; n^a^)	Negative (reports; n^a^)	Mixed (reports; n^a^)	Perceived positive influences	Perceived negative influences
General use12 articles n = 131887	2;37942	7; 93155	3; 790	-Support-Information regarding help seeking-Discussion about mental health	-Normalisation self harm behaviours-Facilitated spread of information and linked otherwise unconnected suicides in a probable suicide cluster
Internet addiction 7 articles n = 42894	0; 0	5; 24873	2; 18021	-Low levels of loneliness and high levels of life satisfaction in individuals with possible internet addiction-Potential protective influence of low levels of internet use compared with no internet use	-Significant relationship between internet addiction and self-harm/suicidal behaviour found in all studies
Sources of help 6 articles n = 14620	4; 142	0; 0	2; 14478	-Successful administration of an online monitoring tool for depression and suicidality-Successful administration of cognitive behaviour therapy and program of treatment for depression -Easily accessed therapy	-Responders to distressed emails more likely to try and solve problems personally than suggest seeking professional help-Individuals with relevant personal histories more likely to report discomfort following questionnaire completion related to self-harm and suicidal behaviours online but it also caused them to think more deeply about their lives
Social media 4 articlesn = 2414	0; 0	3; 1414	1; 1000	-Increasingly used by young people to communicate distress prior to hospital attendance for self-harm.	-Glorification and normalisation self-harm
Forums 14 articles^b^ n = 572	9; 107	0; 0	5; 465	-Isolation reduction -Community and source of support.	-Encouragement to go ahead with suicide plans-Detailed suggestions of suicide method to use-Validation of reasons given for planning suicide
Self-harm website (2 articles; n = 400)	0; 0	1; 71	1; 329	-Use of websites to find help	-Normalisation and reinforcement of self-harm
Video/ image sharing (4 articles; n = 152)	0; 0	1; 0	3; 152	-Factual and educational -Raising awareness for LGBT suicides -Viewing of self-harm images acts as an alternative or deterrent to self-harm	-Comments on videos may serve to maintain or reinforce the behaviour through regular viewing-Comments on videos rarely mention recovery-Explicit imagery of self harm acting as a trigger-Sense of competition
Blogs (2 articles^c^; n = 11)	0; 0	2; 11	0; 0	-No positive influences reported	-Sharing of potentially harmful information including means of concealment of self-harm and suicide methods

a: number of independent participants, i.e. participants contributing to more than one paper are only counted once

b:includes 5 articles related to the same self-harm forum (Sharptalk)

c: includes two articles based on the same set of eating disorder blogs

### General internet use

Twelve studies examined the influence of general internet use [3, 20, 26–35]. Papers were categorised as examining general use if they spoke generally about internet use, did not detail the type of internet use or examined a number of different mediums. Two studies identified positive influences [27, 28]. One of high quality utilised digital to metrics to demonstrate high levels of engagement reported in online discussions about mental health by young people [27]. The second, a low quality study, utilised a community survey and showed lower levels of depression associated with frequent video game use. No association was found between media use and suicidal ideation [28].

Seven studies identified negative influences [3, 26, 29–33]. Studies rated as high quality utilised survey data and showed that high internet users and non-internet users were at higher risk of suicidal ideation and attempted suicide when compared with moderate internet users [31]. An ‘invisible risk’ group of individuals, who spent a lot of time online but did not engage in other risky behaviours (e.g. smoking) and had similar prevalence of suicidal thoughts as the ‘visible’ risk group, was identified in another high quality school based survey [29]. A relationship between internet use and self-harm was reported in several high quality papers. 15% of girls and 26% of boys reported that either the internet or social media had influenced their self-harm [33]. Risky online behaviours such as having a close relationship with someone met online (38% of those who reported self-harm compared with 10% of those who did not) was reported in a set of telephone interviews [32]. This relationship was further supported by an additional low quality school-based survey where suicidal ideation was found to be significantly associated with accessing suicide or self-injury information online (OR 5∙11; 95% CI 0∙35–0∙75), anxiety about getting email replies (OR 2∙06; 95% CI 1∙33–3∙20) and hurtful experiences online (OR 1∙71; 95% CI 1∙03–2∙84) [30]. In addition to the relationship between online behaviours and self-harm it was found that the internet and social media had facilitated the spread of information in a potential suicide cluster, and may have linked suicides otherwise unconnected by school or district [3]. Furthermore, a relationship between internet searches for specific suicide methods and the suicide rate in young people was found in one low quality study [26].

Three studies showed mixed influences [20, 34, 35]. In one small but high quality set of structured interviews participants found some interactive media supportive, while use of violent methods of self-harm were often preceded by viewing suicide related content online [34]. In a medium quality study aiming to examine self-harm content across internet mediums, large dedicated self-harm social media groups, 2290 videos related to self-harm on YouTube and high levels of views of videos about self-harm (the top video being viewed 339646 times) were identified. Peer-driven websites were found to contain warnings of triggering content, including stories and image galleries (not always present). These sites were accessed more frequently than professionally-driven websites [20]. Participants partaking in structured interviews reported that online sources of information related to suicide were quite common, and while social networking sites were frequently cited as sources these were not linked to increases in suicidal ideation, whereas online discussion forums were [35].

### Internet addiction

Seven studies [36–42] examined the relationship between internet addiction and self-harm. For the purpose of this review internet addiction was distinguished from general internet use if papers specifically referred to internet addiction or pathological internet use. There is no agreed definition of internet addiction in the current literature. It ranges from an impulse control disorder likened to pathological gambling [36–38, 41] to assessments of level of functional impairment or level of use [39, 40, 42]. All seven studies were rated as of high or medium/high quality, utilised school-based surveys with validated outcome measures and all found a significant relationship between internet addiction and self-harm/ suicidal behaviour. However, the direction of causality remained unclear. Five of these studies found exclusively negative results [36–40]. When a pathway model was employed problematic internet use was found to predict suicidal ideation alongside suicidal ideation predicting problematic internet use [40]. Two studies showed mixed effects [41, 42]. Potential mechanisms of positive influences included low levels of loneliness and higher levels of life satisfaction in individuals with possible internet addiction and a potential protective influence of low levels of internet use compared with none [42] (Table 3).

### Online intervention/treatment

Six studies were of help administered online with the aim of reducing self-harm/suicidal behaviour, often alongside other manifestations of psychological distress such as depression and anxiety [43–48]. Participants came from a range of settings including schools [43], healthcare settings [44, 46] and universities [47, 48]. Positive influences were identified in four small studies. The successful administration of a program of treatment for depression was demonstrated in one high quality study [46]. Medium quality studies showed the successful implementation of an online monitoring tool for depression and suicidality [44], and for cognitive behaviour therapy [43]. All three of these articles showed a reduction in either self-harm or suicidal ideation. The fourth low/medium quality paper demonstrated that participants positively viewed the idea of mental health services delivered online via questionnaire data and structured interviews [45]. Two much larger studies rated as high [48] and medium quality [47] showed mixed influences, and in the first of these participants did not report negative experiences following the completion of online surveys related to self-harm Participants responding to distressed emails solved problems personally rather than suggesting professional help, with females being more likely to offer help than males [47].

### Social media

Four studies examined the role of social media, including one content analysis paper [21, 24, 49, 50]. For this review social media studies were defined as studies focused on social networking sites such as Facebook and Twitter. Three studies showed negative influences, the studies being rated as low [21, 49] or low/medium quality[24]. It was found that young people attending emergency departments are increasingly using social media to communicate distress prior to hospital attendance for self-harm, particularly to a peer rather than to an adult [49]. Content analysis of open self-harm groups on Facebook revealed glorification and normalisation of self-harm [21]. While Facebook is a moderated site, groups are not moderated in the same way as support forums, where there are often rules on appropriate content. Analysis of comments on MySpace revealed suicidal thoughts, behaviours and intentions indicating significant amounts of hopelessness and despair [24].

One article rated medium/high quality reported the results of an internet survey (n = 1000) and contained mixed results [50]. Tweeting ‘want to die’ or ‘want to commit suicide’ was significantly related to suicidal ideation and behaviour. However, no association was found with simply having a twitter account or tweeting frequently [50].

### Forums

Fourteen articles [15–19, 51–59] related to forums (five content analysis articles). For this review forum use was defined as the use of dedicated support forums, separate from social media sites. The setting of five of these was the ‘Sharptalk’ forum purposely built for research [53–57]. Each article examined a different aspect of forum use, was of low or medium quality and reported a positive influence. There was some reluctance on the part of health professionals in this study to participate actively in forums due to a reported lack of confidence and concerns relating to workload and duty of care [54]. In total, nine papers (five independent studies) reported positive results; one high quality content analysis [16] and four medium and low quality small studies [15, 51–57]. Five studies reported both positive and negative results; one high quality [58] and four medium and low quality [17–19, 59]. All the studies recruited via dedicated discussion forums. One study gathered data via in-depth email interviews [51], all but one of the other papers analysed the content of posts and four studies additionally collected data via questionnaires [53–59]

Positive influences identified in high quality papers include the potential for isolation reduction and informal support [16], and a significant reduction in suicidal thoughts following forum use, although it was noted that a causal relationship could not be directly inferred [58]. Well-moderated self-harm forums appeared to be viewed positively by young people as an online community providing continued support. One study (n = 20) found that levels of forum involvement were associated with lower levels of distress, but this level of distress did not decrease over the three month study period [52]. The potential for support and isolation reduction was supported by a number of small medium and low quality papers [15, 16, 18, 19, 51–57, 59].

Negative influences include the use of forums for destructive means, such as finding a suicide partner and exchange of potentially harmful information found in one high quality paper [58] and four low and medium quality papers [17–19, 59]. Direct encouragement to go ahead with suicide plans, including detailed suggestions of method, and validation for reasons given for a planned suicide were also reported [19]. While some if the discourse was judged to be potentially harmful, no association was found between worsened suicidal thoughts and forum use [59].

### Website with suicide/self-harm content

Two articles reported on websites with dedicated self-harm/suicide content. A medium quality content analysis study of websites indicated negative influences, including that dedicated self-harm websites may normalise and reinforce self-harm [25]. A large high quality cross-cultural survey reported that individuals use these websites to gain help and support. While some participants reported a reduction in self-harm a small number reported increased self-harm associated with these websites.

### Video/image sharing

Four articles focussed on video/image sharing [10, 22, 23, 61]. One medium/high quality content analysis of comments on self-harm videos identified negative influences [22]. Comments on such videos illustrate a strong motivation for sharing experiences online, rarely mention recovery and may contribute to the maintenance of self-harm. The other three papers were of medium and low quality and identified mixed results [10, 23, 61]. Videos with self-harm content on YouTube were found to frequently contain explicit imagery with factual and educational tones [10]. Videos raising awareness of lesbian gay bisexual or trans (LGBT) suicides highlighted that difficulties in life can be overcome; however, negative connotations were also present [23]. Some participants recruited from an online self-harm community reported that viewing of self-harm acted as a deterrent or alternative to self-harm, whilst others reported a triggering effect and sense of competition [61].

### Blogs

Two articles reported the content analysis of the same set of pro-anorexia blogs [62, 63]. Both were rated as low quality and reported negative influences in the form of sharing potentially harmful information related to self-harm and suicide methods and means of concealment.

Across different mediums several studies focused on groups often hidden from traditional research and service provision. For example, three studies focused on populations of LGBT young people [15, 18, 23]. The first was an analysis of the content of YouTube videos raising awareness of the level of suicide in LGBT youth [23]. The other two papers showed that online discussion forums had the potential to engage this hard to reach group and to produce novel, unmediated data [15]. This approach was utilised to gather data on LGBT young people’s perspective on seeking help for suicidal feelings. LGBT individuals found it challenging to articulate emotional distress and seek help from family members or professionals. They were most comfortable communicating online, particularly in dedicated LGBT forums [18]. A further two papers focused on young people with eating disorders [62, 63]. In these two studies a set of eating-disorder blogs were analysed, which showed that discussion of self-harm and suicidal thoughts was common.

## Discussion

This systematic review is an update of a previous smaller review exploring the relationship between internet use and self-harm [5] and incorporates previous evidence, thus also updating the field. A total of 51 articles (representing 46 independent studies) were included, with 192950 individual participants, together with some reports of content analytical studies. While a comparable number of articles included positive (15), negative (19) and mixed (17) influences of the internet on self-harm behaviour, articles demonstrating negative influences included more participants (n = 119524) than those with positive (n = 38191) and mixed influences (n = 35235). On balance, considering the quality of studies and numbers of participants assessed, there is significant potential for harm of online behaviour in relation to self-harm and suicidal behaviour, but also potential benefits that merit exploitation.

Results of this review are largely supportive of the findings of the previous review with comparable proportions of studies with positive and negative results [5]. However, in recent years this field of research has developed substantially allowing greater examination of details such as medium of internet use and for a greater range of positive and negative influences to be identified. Studies on general internet use, internet addiction and online interventions/treatment presented the strongest evidence, with mostly high or medium quality research. High internet use and internet addiction appear to have largely negative influences. Twelve studies examined general internet use, half of which were of high quality. The latter group of studies demonstrated that high levels of internet use (more than two [31] or five [29] hours per day) were associated with suicidal ideation [29, 31]. Further low and medium quality research suggested that self-harm and suicidal ideation were related to searching online for suicide information [30] and that searches for specific methods were related to rates of suicide in young people [26]. Online media appeared to facilitate the spread of information, linking otherwise unconnected suicides, making it difficult to recognize and manage a suicide cluster [3]. Research examining internet addiction represented the most homogenous group of studies; all employed high/medium quality quantitative methodologies in the form of cross sectional school-based surveys and validated outcome measures. All studies examining internet addiction found a relationship between internet addiction and self-harm or suicidal behaviour. The direction of causality of the negative influence of internet addiction and self-harm/suicidal behaviour was unclear. Positive influences included lower levels of loneliness [41] and a potential protective influence of low levels of internet use when compared with no internet use at all [31, 42]. Other studies have found that low and high internet use is associated with higher adolescent health risks than moderate use [31]. The internet may provide an opportunity to be part of a community online when this is lacking in everyday life. The potential of the internet as a medium to deliver interventions to address suicidal behaviours and self-harm was examined in six studies of medium to high quality, with mixed results but generally being viewed positively by participants.

Studies exploring the other mediums (social media, forums, videos/ images sharing, blogs) were smaller, of lower quality and with more mixed results. Only one paper related to social media was rated as medium/high quality. Distressed online posts were found to be related to suicidal ideation and behaviour, but there was no evidence suggesting that simply using social media presents a risk [50]. The remaining low and medium/low quality research found that young people are increasingly using social media to communicate distress, particularly to peers [49]. This is in keeping with more recent research showing that self-harm and suicide-related internet use prior to ED attendance is higher among children and young people than in adults, and is additionally related to higher suicidal intent [64]. While social media is being utilised here to communicate distress, this does not indicate a causal relationship. Glorification and normalisation of self-harm was found in two of the four social media studies [21, 24]. Forums were viewed positively as a source of peer support. There was evidence of a reduction in suicidal thoughts following forum use in one high quality study [58] but also evidence of normalisation of self-harm, encouragement to go ahead with suicide plans and discussion of how to conceal self-harm, supported by one high quality and a number of medium and low quality studies [16, 17, 19, 50, 58]. The impact of forum use on levels of self-harm remains unclear. Videos were highly viewed/shared, largely factual or educational and often contained graphic imagery, but rarely with warnings of such content [10]. One high quality study found that comments on videos may contribute towards the maintenance of self-harm and suggests a strong motivation for sharing experiences online. It remains unclear whether this sharing of experiences has a positive or negative impact on individuals, with its influence likely to vary with individual circumstances [22]. In the remaining (low quality) study some participants reported an alleviating effect of images, others a triggering one [61]. Sharing of potentially harmful information related to self-harm and suicide was reported in two small low quality studies of the same set of pro-anorexia blogs [62, 63].

The variation in results between mediums may be partially attributable to study design and participant samples. Research examining general internet use and internet addiction was largely based on school surveys employing validated outcome measures and found largely negative influences of internet use. In contrast, research examining forum use recruited almost exclusively from online discussion forums, undertook content analysis of forum posts, sometimes alongside questionnaire data, and often found positive influences. The contrasting results between studies recruiting from general population and self-selecting participants are not unexpected. Studies with more diverse participant samples and employing validated outcome measures may assist in clarification of the effects of different types of medium.

Research in this area may be biased towards measuring certain effects and outcomes e.g. a positive effect for interventions, or a negative effect for internet addiction. The choice of outcome measures may reflect the expected effects of the internet and fail to capture the full complexity of the experience of individuals. This is a risk in all research but may be a particular issue in this field. For example, studies examining internet addiction report largely negative results. However one study found that internet addicted individuals reported high levels of life satisfaction and low levels of loneliness that may not have been identified had these measures not been included [41]. In future investigations researchers should aim to ensure that outcome measures capture both possible positive and negative outcomes in order to give a complete and unbiased picture. Such steps can be seen in more recent research. For example an online treatment for depression resulted in symptom reduction but examination of possible negative influences demonstrated that individuals with lower education were at higher risk of symptoms becoming more severe [65]. Another recent study found both positive and negative influences of websites detailing suicide methods [66].

In keeping with the previous review [5] the majority of studies finding solely negative influences utilised quantitative methodologies. Mixed methods and qualitative studies tended to report more mixed results. It is unclear if this is a discrepancy between what participants report and actual outcomes or if quantitative data are failing to capture the complexity of the issue. This is further compounded by the mostly low quality of included qualitative studies. High quality qualitative and quantitative research is needed to establish whether differences in the influence of various mediums are partially attributable to study design.

### Strengths and limitations

The potential for publication bias exists in any review of literature and should be considered when interpreting the results. Steps were taken to minimise any bias as much as possible including conducting an extensive search of multiple databases including grey literature databases and topic specific websites, reviewing reference lists and contacting experts in the field. However, only English language publications were included. It was noticeable that males remain under-represented in studies. Further examination of any gender differences is a potential avenue for future research.

While the decision to review literature related to cyber-bullying in a separate review will have had an influence on the proportion of articles reporting positive and negative results, it will allow for a more in-depth discussion of this important topic. The inclusion of this large body of research would have created an unwieldy review or necessitated a more cursory discussion of internet mediums. Literature related to cyber-bullying is unlikely to report any positive outcomes and had it been included it would have added to studies reporting a negative influence. This could potentially be viewed as an important bias in this review. However, results of this review highlight that the internet has the potential for both positive and negative influences dependent on the way in which it is used. Identifying both beneficial and harmful mediums and directing individuals towards healthy online behaviour should be considered of greater importance than weighing up whether the internet is simply ‘good’ or ‘bad’.

While this review summarises data from an extensive search, there will be further literature published since the search was conducted. Based on the number of additional articles in the two years since the review by Daine et al [5] it is likely that this will represent a considerable body of research. Summarising it is likely to be beyond the scope of one review. Because of the rapidly increasing body of research, authors conducting future systematic reviews on this topic may consider reviewing by specific internet medium (forums, videos etc.) to ensure the task is manageable and the resultant reviews are of sufficient depth in each area to allow identification of key messages for clinicians and policy makers. While a range of internet mediums were included in this review, recent research has expanded even further, for example examining behaviours such as online gambling [67].

The quality of qualitative studies in this review appeared mostly to be low. While this may be reflective of quality, the appropriateness of using checklists to assess qualitative research has been questioned due to the diversity of approaches in collecting, analysing and interpreting data [68, 69]. This may be particularly problematic with research into internet use where there is considerable heterogeneity in methodology across study designs. It is unlikely that a single checklist would be suitable to capture all aspects of study quality across the range of study designs, populations and outcomes. Authors of future reviews in this area might consider the selection of quality outcome measures that fully capture the quality of various study designs.

It is not always possible in studies examining forum use or comments on videos/photos to accurately determine the characteristics of individuals and young people may misrepresent their age when creating profiles. Therefore ages of participants were not always clearly reported but were inferred by study authors based on demographics of typical users of sites or based on profile information.

It was a challenge to categorise findings as negative or positive in some studies [5]. The sharing of experiences online, for example, while therapeutic for some, may be destructive for others. The expression of distress online could be viewed as negative on the one hand, but as an opportunity for intervention on the other. This is an area of research where outcomes are not always clear cut. It was not always easy to understand whether certain factors acted as mediators or moderators of distress, nor the long-term implications. For example, individuals with a history of self-harm were more likely to report discomfort following online questionnaire completion related to self-harm, but it also caused them to think more deeply about their lives which may have been positive [48]. While it has been possible here to broadly discuss the influence of various mediums and to identify those that may represent a higher risk, the impact of various aspects of internet use is likely to vary between individuals and over time and should therefore always be assessed on an individual basis.

A wider range of internet mediums was examined than was possible previously [5]. This has resulted in a varied pool of participants, including those recruited from discussion forums, schools and healthcare settings. The majority of quantitative studies employed validated outcome measures, in contrast to literature identified in the previous review [5], allowing some comparison of results across studies. However, the considerable range in methodology, population and outcomes studied means that, at this time, it is not possible to conduct a meaningful statistical meta-analysis.

### Implications

The volume of self-harm videos shared on YouTube and the high number of views and comments have led to suggestions of developing videos to emphasise help and recovery [10, 22]. The introduction of psycho-educational prevention programmes in schools concerning appropriate responses to distressed posts on social media and digital citizenship may mitigate some of the negative influences of the internet [20, 47, 49]. The internet is a potential tool for outreach by health professionals. Research suggests some disconnect between healthcare professionals and media usage [11]. This was only explicitly discussed in one set of studies reviewed here in which health professionals expressed discomfort about engaging with young people in an online setting and had concerns over duty of care [54]. This could be addressed through further training and encouragement of clinicians working with young people who self-harm or have mental health issues to engage in discussion about internet use. This should be a standard item during assessment. It could include the asking about the role of images/videos [61] and designing treatment plans to maximise beneficial online behaviours and reduce associated harms [20].

Suggestions have been made for the implementation of guidance to individuals and service providers such as avoiding details of method and including warnings of graphic content on web pages [20]. Stricter regulations could be modelled on the initiative in Australia, where pro-suicide sites were banned in 2006 [70]. Several major social media platforms (Tumblr, Pintrest, Instagram, Facebook) have responded to concerns and implemented policies regarding posts related to self-harm. Such content may not be searchable, is banned or brings up links to counselling and prevention resources [71]. The potential to access groups online largely hidden from the health service, such as those for LGBT individuals or those with eating disorders for both interventions and research may improve access to care and allow representation in research that has not been possible previously. Any online sign-posting or interventions should not be limited to platforms exclusively dedicated to self-harm but should also extend to other groups at potential risk. This is supported by recent research finding that experiences of victimisation are associated with entering pro self-harm and pro-suicide websites [72].

### Conclusions

Research concerning the internet and self-harm in young people is rapidly evolving in an attempt to keep pace with the continually changing nature of its use. On balance, considering the quality of studies and numbers of participants assessed in this review, there is significant potential for harm to result from online behaviour in relation to self-harm and suicidal behaviour (normalisation, triggering and competition between users, a source of contagion and harmful information for vulnerable individuals), but also the potential to exploit its benefits (a sense of community, crisis support and reduction of social isolation). The focus should now be on a range of internet mediums including social media, video/image sharing, and the potential for the internet to be used in therapy and recovery.

There were a number of innovative suggestions from research teams responsible for publications, including educational programmes in schools to teach young people how to respond to distressed posts/messages on social media and repeated calls for clinicians to be aware of internet use. The internet may also represent an under-utilised setting to access ‘hidden’ at risk groups, giving them a voice both in research and in practice.

## Supporting information

S1 Table Sources searched and terms used. (DOCX)

S2 Table Data extraction sheet. (DOCX)

S3 Table Quality scores by study design. (DOCX)

S4 Table Outcomes studied and measures used. (DOCX)

S5 Table PRISMA 2009 checklist. (DOC)
