# Correction: A systematic review of the relationship between internet use, self-harm and suicidal behaviour in young people: The good, the bad and the unknown

**DOI:** 10.1371/journal.pone.0193937

**Published:** 2018-03-01

**Authors:** Amanda Marchant, Keith Hawton, Ann Stewart, Paul Montgomery, Vinod Singaravelu, Keith Lloyd, Nicola Purdy, Kate Daine, Ann John

There are errors in Tables 1 and 2 and Supporting Information S3 and S4 Tables. The studies Robertson, 2012 [3], Dunlop, 2011 [35], and Sueki, 2015 [50] are incorrectly classified due to a merging error in earlier drafts. Please see the corrected Tables 1 and 2 and Supporting Information S3 and S4 Tables below.

**Table 1:** Summary of included studies.

Internet medium	Lead Author, year, country	Population (N, %female)	Aims, Objectives	Results, outcome	Outcome	Design, Quality score
**General use**	Carew, 2014 [27], Canada and USA	Internet users (28805; 64)	To investigate mental health information seeking online, and to identify differences within age groups and geographical location	A 200% increase in online activity regarding mental health was identified (between 2006 [baseline] and 2010). Adolescents were most likely to initiate conversation about depression followed by anxiety, alcohol, suicide, sexting and marijuana. Adolescents tended to discuss concerns through the use of personal stories.	Positive	Quantitative High
	Casiano, 2012 [28],Canada	Canadian young people aged 12–19 (9137; 49)	To examine to association between quantity of media use and health outcomes in adolescents	No significant association between any form of media and suicide ideation (internet use OR 0.98, 95% CI 0.83–1.16)	Positive	QuantitativeLow
	Carli, 2014 [29], 11 European countries	School based adolescents in eleven European countries (12395; 55)	To explore the prevalence of risk behaviours (excessive alcohol, drug use, truancy etc.) and their association with psychopathology and self-destructive behaviours	Latent class analysis identified three groups of adolescents: high risk, including pupils who scored high on all risk behaviours; low risk including pupils with low frequency of behaviours and invisible risk. This ‘invisible risk’ group was found to score high on use of media and have similar prevalence of suicidal thoughts/psychopathology as ‘visible risk’ group. The invisible risk group were at significantly higher risk than the low risk group for non-suicidal self-injury (Relative risk ratio (RRR) = 1.40; 95% CI 1.13 1.84), suicidal ideation (RRR = 1.29; 95% CI 1.12–1.48) and suicide attempt (RRR = 1.22; 95% CI 1.22–2.35).	Negative	QuantitativeHigh
	Hagihara, 2012 [26], Japan	Young adults in Japan; Rate of suicide;	To examine the association between suicide-related searches and the incidence of suicide on young adults in Japan	Association between Internet suicide-related searches and the incidence of suicide in Japan (over 77 months): the terms "hydrogen sulphide", "hydrogen sulphide suicide", and "suicide hydrogen sulphide suicide" at (*t*-11) were related to the incidence of suicide among people aged in their 20s (*P* = 0.005, 0.005, and 0.006, respectively).	Negative	Quantitative Low
	Katsumata, 2008[30], Japan	Japanese high school students (590;49)	To investigate the association between the experience of using electronic media and suicidal ideation in Japanese adolescents.	Suicidal ideation was significantly associated with anxiety about not getting email replies (OR 2.06; 95% CI1.33–3.20), and searching online for information about suicide and self-harm (OR 5.11; 95% CI 2.43–10.71) and hurtful experiences on the web (OR 1.71; 95% CI 1.03–2.84)	Negative	QuantitativeLow
	Kim, 2012 [31], Korea	Korean middle and high school students (75066; 47)	To consider the association between internet using time for non-educational purposes and adolescent health	Internet non-users (NIU) and heavy internet users (HIU) were found to be high risk groups when compared with moderate internet users (MIU) on multiple mental health measures. Suicide ideation was significantly higher in HIU and NIU (females: HIU = 43.4%; NIU 25.8%; OIU 21.8% (P<0.001); males: HIU 26.4%; NIU 16.7%; OIU 13.6% (P<0.001)) as was rate of attempted suicide (females: HIU 13.9%; NIU 7.3%; OIU 5.2% (P<0.001); males: HIU 10%; NIU 4.9%; OIU 2.4% (P>0∙001))	Negative	Quantitative High
	Mitchell, 2007 [32], USA	American internet users aged 10–17 (1500; 81)	To explore internet use and interpersonal interactions of youth reporting self-harm	Youth who self-harm engaged in more risky online behaviours than those who did not including using chat rooms (57% compared with 29%) and to have a close relationship with someone they met online (38% vs. 10%)	Negative	QuantitativeHigh
	O’Connor, 2014 [33], Northern Ireland	Adolescents in Northern Ireland (3596; 48)	To determine the prevalence of self-harm and associated factors	Self-harm was found to be associated with internet/social media as well as variety of other factors including exposure to the Northern Ireland conflict. In total 15% of girls and 26% of boys endorsed either the internet or social networking sites as factors that influenced their self-harm.	Negative	Quantitative High
	Robertson, 2012 [3], New Zealand	New Zealand adolescents (8; 88)	To describe an adolescent suicide cluster and the possible role of online social networking and text messaging as sources or contagion and obstacles to recognition of a potential cluster	These cases did not belong to a single school but were linked by social networking sites including memorial pages. This facilitated the rapid spread of information and made recognition and management of a possible cluster more difficult	Negative	Mixed methodsMedium
	Collings, 2011 [34], New Zealand	New Zealand adolescents (71; 79)	To describe the influences of media on suicidal behaviours, from the perspectives of young people.	Participants considered some interactive media supportive. 80% (n = 12) of those who used violent methods of self-harm had been exposed to suicide content via the internet before the incident	Both Positive and negative	Mixed MethodsHigh
	Duggan, 2012 [20], Canada	Scope and nature of self-harm content across various internet mediums	To examine the scope and nature of self-harm content across informational/interactive websites, social networking websites and YouTube	Results suggest that peer driven websites are accessed more often than professionally driven websites. Self-harm is strongly represented among social networking websites and YouTube evidenced by large group memberships and video counts. The search terms yielded 41 dedicated groups on Facebook with memberships ranging from 2 to 4,686. The same search yielded 206 groups on MySpace with group membership ranging from 2–1653. Searches on YouTube produced 2,290 videos. Characteristics of groups, videos and posters are described.	Both positive and negative	QuantitativeMedium
	Dunlop, 2011 [35], USA	Young people aged 14–24 (719)	To determine whether online news and social networking sites, expose young people to suicide stories that might increase suicide ideation	Online sources of information were quite common (reported by 59% of participants). Social networking sites were frequently cited as sources but were not linked to increases in ideation. However online discussion forums were associated with increases in suicide ideation	Both positive and negative	Quantitative High
**Internet addiction**	Kaess, 2014 [36],11 European countries	School based adolescents in eleven European countries (11356; 57)	To investigate the association between pathological internet use, psychopathology and self-destructive behaviours	Suicidal behaviours, depression, anxiety, conduct problems and hyperactivity/inattention were significant and independent predictors of pathological internet use (Suicidal ideation coefficient 0.324, 95% CI 0.251–0.397, P <0.001; Suicide attempts coefficient 0.552, 95% CI 0.207–0.896, P = 0.001). This association is significantly influenced by country and gender.	Negative	QuantitativeHigh
	Kim, 2006 [37], Korea	High school students in Korea (1573; 65)	To elucidate the relationship between internet addiction, depression, and suicidal ideation	Internet addiction scores were positively correlated with suicidal ideation in non-internet addicts, possible addicts and internet addicts (non-addicted r = 0.111, p = 0.001; possible addicted r = 0.147, p < 0.001; internet addicted r = 0.448, p<0.001)	Negative	QuantitativeHigh
	Lam, 2009 [38], China	Adolescents in china (1639;55)	To examine the association between internet addiction and self-harm	Moderately or severe internet addiction was associated with higher incidences of self-harm (adjusted OR 2.0, 95% CI 1.1–3.7).	Negative	Quantitative High
	Lin, 2014 [39], Taiwan	Taiwanese adolescents aged 12–18 years (9510; 52)	To examine the associations of suicidal ideation and attempt with internet addiction and activities	Internet addiction was significantly associated with suicidal ideation (OR 1.25, 95% CI 1.08–1.44) and suicide attempt (OR 1.59, 95% CI 1.29–1.96). Specific internet activities associated with increased and decreased risk	Negative	QuantitativeHigh
	Park, 2013 [40], Korea	Korean middle and high school students (795; 68)	To evaluate a)associations between problematic internet use and depression, bipolar disorder symptoms and suicidal ideation; and b) whether mood disorders mediate the relationship between suicidal ideation and problematic internet use	Presence of problematic internet use significantly associated with suicidal ideation (OR = 5.82, 95% CI = 3.30–10.26, p<0.001) as well as depression (OR = 5.0, 95% CI = 2.88–8.66, p<0.001) and probably bipolar disorder (OR = 3.05, 95% CI 0.96–9.69, p = 0.059). Problematic internet use was found to predict suicidal ideation (ß = 0.115, 95% CI = 0.052–0.193, p = 0.006). Conversely suicidal ideation was found to predict problematic internet use ((ß = 0.215, 95% CI 0.089–0.346, p = 0.006). Complex transactional relationship.	Negative	QuantitativeMedium/high
	Aktepe, 2013 [41], Turkey	High school students in Isparta (1897; 43)	To measure the prevalence of internet addiction and to detect related socio-demographic factors	The prevalence of possible internet addiction was found to 14%. A significant association between problematic internet use and self-harm was found (ß = 0.574, OR = 1.79, 95% CI 1.30–2.43, P <0.001). Adolescents with possible internet addiction were also found to have low levels of loneliness and high levels of life satisfaction.	Both positive and negative	QuantitativeHigh
	Messias, 2011 [42], USA	Students aged 14–18 years; (16124; N/A)	To investigate the association between excessive video game/internet use and teen suicidality	Teens who reported more than 5 hours a day of video game/internet use had a significantly higher risk of suicidal ideation (OR = 1.7, 95% CI 1.3–2.1) and suicide planning (OR = 1.5, 95% CI 1.1–1.9). Authors find a potential protective influence of low video game use compared with no use.	Both positive and negative	QuantitativeHigh
**Sources of help**	Hetrick, 2014^a^ [43], Australia	Melbourne high school students experience suicidal ideation; (21)	To investigate the usefulness of an internet- based CBT programme	Over the course of the intervention negative problem-solving orientation improved (t = 4.38, p < 0.0005) and students relied less on emotion focused coping strategies. Adolescents rated the problem-solving and cognitive restructuring modules as particularly helpful.	Positive	QuantitativeMedium
	Hetrick, 2015 [44], Australia	Australian young people aged 15–24 (15)	To develop and examine the feasibility of an online monitoring tool of depression symptoms, suicidality and side effects	Results show that an online monitoring tool is potentially useful as a systematic means for monitoring symptoms of depression and suicidality, but further research is needed including how to embed the tool within clinical practice	Positive	Mixed methodsMedium
	Mar, 2014 [45] UK	Individuals age 16–24 who had experienced suicidal ideation (23; 96)	To explore youth consumer preferences for online interventions targeting depression and anxiety	Youth positively received the idea of e-mental health services. Noted preferences for services that are simple to use, interactive and include support through an online community.	Positive	Mixed methodsLow/medium
	Saulsberry, 2013 [46], USA	Adolescents screening positive for depression in primary care (83; 57)	To test an internet program for young people with depression	Participants demonstrated significant within-group decreases in depression and self-harm ideation (any thoughts of self-harm in previous two weeks 14.46% at baseline compared with 4.82% at 1 year follow-up)	Positive	QuantitativeHigh
	Barton, 2013 [47], USA	College students (106; 55)	Study examined responses to open-ended email vignettes from a fictitious friend exhibiting depressed, irritable or suicidal communications	Results indicate student’s preferences for solving fictitious peer problems personally rather than professionally. Patterns of help-giving and sex differences varied by condition	Both positive and negative	QualitativeMedium
	Whitlock, 2013 [48], USA	College students (14372; 43)	To examine the impact of questions regarding self-injury, suicide and psychological distress in a web-based survey on respondents	Less than 3% of individuals reported negative survey experiences. Individuals with relevant personal experience reported greater discomfort with the survey yet were also significantly more likely to report that it caused them to reflect on their lives	Both positive and negative	Mixed methodsHigh
**Social media**	Belfort, 2012 [49], USA	Adolescents presenting to hospital with self-harm (1350; 75)	To describe key similarities and difference among adolescents who communicated their suicidality via electronic Vs. other means	Numbers of electronic communication of suicidality increased over time from 8.3% in 2005 to 55.6% in 2009. Patients who communicated suicidality electronically more likely to do so to a peer (67% compared with 7% of those communicating by other means).	Negative	QuantitativeLow
	Cash, 2013 [24], USA	MySpace users aged 13–24 (64; 40)	To explore the ways in which adolescents use MySpace to comment on their suicidal thoughts and intention	Comments referenced a significant amount of hopelessness, despair and desperation. Adolescents use public web sites to display comments about their suicidal thoughts, behaviours and intentions.	Negative	QualitativeLow/medium
	Zdanow, 2012 [21], South Africa	Analysis of self-harm groups on Facebook	To analyse the representation self-harm on dedicated Facebook groups	Content analysis of two groups revealed glorification and normalisation suicidal behaviours. Potential for social networking sites to be used as a tool for the promotion and encouragement self-harm	Negative	QualitativeLow
	Sueki, 2015 [50], Japan	Young adult twitter users (1000; 61)	To examine the association between suicide-related tweets and suicidal behaviour to identify suicidal young people on the internet	Logistic regression analysis showed that tweeting ‘want to die’ was significantly associated with history of suicidal ideation (OR = 2.53, 95% CI 1.61–3.99) having a suicide plan (OR = 2.55, 95% CI 1.56–4.17) and attempting suicide (OR = 1.67, 95% CI 0.95–2.94). Tweeting ‘want to commit suicide’ was significantly related to history of self-harm (OR = 1.87, 95% CI 1.03–3.41), having a suicide plan (OR = 1.92, 95% CI 1.07–3.46) and attempting suicide (OR = 3.48, 95% CI 1.89–6.42). Having a twitter account and tweeting daily were not associated with suicidal behaviour	Both positive and negative	QuantitativeMedium/High
**Forum**	Baker, 2008 [51], UK	Users of self-harm discussion forums (10, 50)	To explore the accounts of young people who self-harm and use forums	Forums were used positively for support and communication. Some participants report a reduction in the incidence of self-harm	Positive	QualitativeLow
	Barak, 2006 [52], Israel	Users of self-harm discussion forums (20, 75)	To assess whether the degree of forum involvement affected distress levels	Levels of forum involvement was association with lower levels of distress, however levels of distress did not improve over three months (F = 2.10; df = 2, 787)	Positive	Mixed MethodsLow
	Jones^b^, 2011 [53], UK	Users of a self-harm forum built for research (77, 95)	To explore what young people who self-harm think about online self-harm discussion forums	Participants claimed to learn more about mental health issues from online forums than from information sites, find it easier to talk about self-harm to strangers than to family or friends and preferred to talk online than in person.	Positive	Mixed MethodsMedium
	McDermott, 2013 [15], UK	Analysis of forum posts	To use qualitative methodology to examine internet forums where LGBT^c^ youth discuss self-harming	This methodology can address some research dilemmas by generating diverse samples and a different type of unmediated complex data. Online data can enhance understanding of hard-to-reach youth	Positive	Qualitative Low/Medium
	Owens^b^, 2012 [54], UK	Users of a self-harm forum built for research (77; 95)	To bring together young people who self-harm and health professionals online	The young people were keen to share their experiences and supported one another during crises. Health professionals did not actively participate in forums due to reported lack of confidence and concerns relating to workload and duty of care.	Positive	Mixed methodsMedium
	Sharkey^b^, 2012 [55], UK	Users of a self-harm forum built for research (77, 95)	To use discourse analysis and the concept of face-work as a framework to understand interactions in a self-harm support forum	Use of a range of mitigation devices found suggesting that the young people orient a ‘protective line’ in their supportive interactions. This may enable a more trusting, open context for support.	Positive	QualitativeMedium
	Smithson^b^, 2011 [56], UK	Users of a self-harm forum built for research (77, 95)	To explore how young adults became members and sustained membership in a self-harm support forum	Participants displayed expectations about appropriate ways of discussing self-harm, responses and advice. Participants were active in shaping interaction on the forums requesting input from moderators.	Positive	QualitativeLow
	Smithson^b^, 2011 [57], UK	Users of a self-harm forum built for research (77, 95)	To investigate the nature of problem presentation and responses in an online support forum	Analysis highlighted the tendency to offer advice where it was not asked for and the mundane ‘safe’ nature of advice	Positive	QualitativeLow
	Whitlock, 2006 [16], USA	Analysis of forum posts	To investigate the prevalence and nature of self-injury forums, to explore the content, role and influence of discussion forums	Informal support was the most common type of exchange (28.3% of posts). Concealment of practice (9.1%), perceived addictiveness (8.9)and formal help-seeking (7.1) were also discussion themes	Positive	Mixed MethodsHigh
	Eichenberg, 2008 [58], Germany	Users of suicide discussion forums (164; 50)	To assess the assumption that suicide message boards are harmful.	Both constructive (e.g. help-seeking) and destructive (e.g. finding a suicide partner) motives were identified. A significant reduction in suicidal thoughts was found following forum use (effect size *d =* 0.72 (t [144] = 9.2; *p* < 0.01). Unable to directly infer cause.	Both positive and negative	QuantitativeHigh
	Franzen, 2011 [17], Sweden	Qualitative study of a Swedish-speaking web community connected to self-harm	To analyse how self-injuring men and women construct themselves as cutters	Two main interdependent discourses are identified within the web community: the ‘normalising’ and the ‘pathologizing’.	Both positive and negative	QualitativeLow
	McDermott, 2015 [18], UK	Analysis of web-based discussions	To utilise qualitative virtual methods to investigate LGBT^c^ youth web-based discussions about seeking help for suicidal feelings and self harming	Young people wanted assistance but found it difficult to ask for help and articulate emotional distress	Both Positive and negative	QualitativeLow/medium
	Sueki, 2012 [59], Germany and Japan	Users of suicide forums in Japan and Germany (301, 54)	To analyse the cross-cultural use of suicide forums in Japan and Germany	Factor analysis demonstrated two motives: mutual help and suicide preparation. Suicidal thoughts did not worsen with forum use and there was no difference in demographics, motives or effects of suicide forums between Germany and Japan	Both Positive and negative	QuantitativeLow/ medium
	Westerlund,2013 [19], Sweden	Analysis of young adult forum posts	To examine conversations about suicide on discussion forums	Most participants communicate based on a need to gain acceptance and understanding. However there was also exchange of suicide methods and encouragement to go ahead with suicide plans	Both positive and negative	QualitativeLow
**Website with suicide/self-harm content**	Lewis, 2011 [25], Canada	Authors and users of self-harm websites (71; 79)	Examination of the content of non-suicidal self-injury web sites	Websites depict self-harm as an effective coping mechanism (92%), addictive (87%) and not always painful (24%). Almost all websites contained melancholic tones (83%) and several contain graphic imagery (30%). Overall it is suggested that such sites may normalize and reinforce self-harm	Negative	QualitativeMedium
	Harris, 2013 [60], Cross cultural (UK Europe, Canada, Australia, New Zealand and others)	Self-selected users of self-harm websites (329; 92)	To explore the reasons people visit self-harm websites or forums; beliefs regarding these sites; how the use of such sites modulates self-harm and other impacts of these sites on the lives of those who use them	65.6% of participants visited sites at least twice a week, 78.2% used sites to find information and 68.4% to participate in forums. Positive effects of website use such as gaining help and support and reduction in self-harm behaviours were reported by a large number of participants. However smaller numbers reported negative effects including worsened self-harm	Both positive and negative	Mixed methodsHigh
**Video/image sharing**	Lewis, 2012 [22], Canada	Analysis of comments on YouTube videos related to self-harm	To examine viewers comments on non-suicidal self-injury YouTube videos and determine potential risks and benefits of such videos	Viewer’s responses to videos may maintain the behaviour with admiration of video quality (21.95%), message (17%) and up-loader (15.40%) common. Comments rarely encourage or mention recovery (<3%). Sharing experiences online is a strong motivator for viewers of self-harm related videos	Negative	QualitativeMedium/High
	Grzanka, 2014 [23], USA	Critical discourse analysis of online videos	To investigate a mass-mediated campaign against a perceived increase in suicides among gay youth in America	Analysis of videos showed a neoliberal frame that places the burden of a ‘better’ life onto youth who are instructed to endure suffering in the interest of inevitable happiness	Both positive and negative	QualitativeLow
	Lewis, 2011 [10], Canada	Posters of self-harm videos on YouTube (100; 95)	To examine the accessibility and scope of non-suicidal self-injury videos online	The top 100 videos were viewed over 2 million times and most were accessible to a general audience. Viewers rated videos highly (M = 4.61; SD 0.61 out of 5.0) and selected videos as a favourite over 12000 times. Explicit imagery common (64% of videos) with many videos not warning about this content	Both positive and negative	Mixed methodsMedium
	Sternudd, 2012 [61], UK, USA Europe	Young people who self-harm (52; 87)	To examine reasons for, and reactions to producing/viewing self-harm images online	Informants reported effects images was alleviating rather than triggering. When interpreting statements about images 40% were positive and 25% were negative. To publish them was a way of sharing experiences with others and to give or receive help. Participants emphasised that the outcome of viewing these photos varies by individual and situation	Both positive and negative	Mixed methodsLow
**Blogs**	Castro^d^, 2012 [62], Portugal and Brazil	Authors of Portuguese language blogs (11, 82)	Analysis of pro-anorexia blogs to systematize and categorize their characteristics, content and messages	Blogs can have negative consequences as a result of sharing harmful information about fasting, drugs, self-harm and suicide	Negative	QualitativeLow
	Castro^d^, 2013 [63], Portugal and Brazil	Authors of Portuguese language blogs (11, 82)	Analysis of pro-anorexia blogs to better understand the influence of social and cultural pressures	Positive relationship found between social and cultural pressures and engaging in self-harming/destructive behaviours	Negative	QualitativeLow

^a.^ Part of a three part series related to online interventions. Subsequent two papers while cited in press have publication dates outside of current search

^b.^ Five reports related to the same self-harm forum study (Sharptalk)

^c.^ Lesbian Gay Bisexual and Transgender

^d.^ Two reports based on the same set of eating disorder blogs

**Table 2:** Research methodology and CASP quality score by internet medium.

Variable		General use (12 papers; n = 131887(papers; n^a^)	Internet addiction (7 papers; n = 42894) (papers; n^a^)	Sources of help (6 papers; n = 14620) (papers; n^a^)	Social media (4 papers; n = 2414)(papers; n^a^)	Forums (14 papers^b^; n = 572)(papers; n^a^)	Self-harm website (2 papers; n = 400)(papers; n^a^)	Video/ image sharing (4 papers; n = 152)(papers; n^a^)	Blogs (2^b^ papers; n = 11)(papers; n^a^^c^)	Total (51 papers; 46 studies; n = 192950)(papers; n^a^)
**Methodology**	**Quantitative**	10; 131808	7; 42894	2; 104	2; 2350	2; 465	0; 0	0; 0	0; 0	23; 177621
	**Qualitative**	0;0	0; 0	1; 106	2; 64	8; 10	1; 71	2; 0	2;11	16; 262
	**Mixed**	2; 79	0; 0	3; 14410	0; 0	4; 97	1; 329	2; 152	0; 0	12; 15067
**CASP quality score**	**High**	7; 122152	6; 42099	2; 14455	0; 0	2; 164	1; 329	0; 0	0; 0	18; 179199
	**Medium**	2;8	1; 795	4; 165	2; 1064	6; 378	1; 71	2; 100	0; 0	18; 2581
	**Low**	3; 9727	0; 0	0; 0	2; 1350	6; 30	0; 0	2;52	2;11	15; 11170

^a^: number of independent participants, i.e. participants contributing to more than one paper are only counted once

^b^: includes 5 reports related to the same self-harm forum (sharptalk)

^c^: includes two reports based on the same set of eating disorder blogs

There are errors in the eighth and ninth sentences of the Results section. The correct sentences are: Using the CASP quality score 18 articles were assessed as high quality, 18 as medium quality and 15 as low (S3 Table). The quality of articles varied by study design with a greater proportion of quantitative (14/23) than qualitative ones (0/16) rated as high quality.

There is an error in the fifth sentence of the second paragraph of the Discussion section. The correct sentence is: Twelve studies examined general internet use, seven of which were of high quality.

## Supporting information

S3 Table Quality scores by study design. (DOCX)

S4 Table Outcomes studied and measures used. (DOCX)
