# Supplementary material for: A systematic review of the relationship between internet use, self-harm and suicidal behaviour in young people: The good, the bad and the unknown
Source: PLoS One. 2017 Aug 16;12(8):e0181722. doi: 10.1371/journal.pone.0181722 (PMC5558917; doi:10.1371/journal.pone.0181722)
Supplement: S1 Table — (DOCX) [file pone.0181722.s001.docx]

**Supplementary Table 1**

**Search Terms Employed For Electronic Literature Search**

**Databases searched**

CINAHL, Cochrane Library, EMBASE (excluding Medline journals), HMIC, Library catalogue and knowledge base, Medline, NICE, Prospero, PsycINFO, PUBMed, SCOPUS. Additional searches were conducted in health improvement sources (Bibliomap, Database of Promoting Health Effectiveness Reviews, Health Evidence Canada, Campbell, EPPI, Health Evidence Network (WHO), NICE pathways), topic specific websites (American Association of Suicidology, British Psychological Society, CEBMH, Centre for Mental Health, DH, DHSPSS-NI, MFH, NHS Scotland, Royal College of Psychiatrists, Welsh Government) and meta-search engines (Google/ Google scholar)

**The following terms were searched in free text/keywords:**

'Automutilation ', 'Distress*', 'Emotion*', 'nssi', '((oneself or myself or self) adj2 (cut* or harm* or hurt* or kill or injur* or mutilat*))', '(psychological adj (stress or distress))', 'SIB', 'Suicid*', 'Aol', 'Askfm', 'Bebo', 'blog*', 'chat room* OR chatroom*', 'cyber*', 'discussion forum', 'e-communi*','e-material*', 'Facebook', 'google*', 'hashtag', 'image sharing', 'Instagram', 'instant messag*', 'Internet*', 'live chat', 'live journal*', 'meme', 'MSN', 'Myspace', 'on line OR online', 'photo sharing', 'Pinterest', 'podcast*', 'social network*', 'spam*', 'troll*', 'Tumblr', 'tweet*', 'Twitter', 'video sharing', 'vine', 'virtual*', 'vlog*', 'web*', 'YouTube'

**Alongside the following database subject headings:**

MESH: ‘Self-Injurious Behavior’, ‘Stress, Psychological’, ‘Blogging’, ‘Electronic Mail’, ‘Internet’, ‘Social media’, ‘Social networking’, ‘Adolescent’, ‘Child’, ‘Students’, ‘Young adult’

HMIC: ‘Attempted suicide’, ‘Self harm’, ‘Suicide pacts’, ‘Suicide’, ‘Cyberspace’, ‘Internet’, ‘internet websites’, ‘intranet’, ‘world wide web’, ‘Children’, ‘Young people’

PsycInfo: ‘attempted suicide’, ‘self destructive behaviour’, ‘self injurious behavior’, ‘suicidal ideation’, ‘suicide prevention’, ‘electronic communication’, ‘internet’, ‘internet addiction’, ‘Online Social Networks’, ‘online therapy’, ‘social media’, ‘Social Networks’, ‘Websites’, ‘Adolescent Attitudes’

Embase:‘automutilation’, ‘suicidal behaviour’, ‘suicide’, ‘email’, ‘internet’, ‘social network’, ‘Adolescent’, ‘child’, ‘young adult’
