# Supplementary material for: A systematic review of the relationship between internet use, self-harm and suicidal behaviour in young people: The good, the bad and the unknown
Source: PLoS One. 2017 Aug 16;12(8):e0181722. doi: 10.1371/journal.pone.0181722 (PMC5558917; doi:10.1371/journal.pone.0181722)
Supplement: S2 Table — (DOCX) [file pone.0181722.s002.docx]

**Supplementary Table 2 Data extraction sheet**

| **Author/Year** |  |
| --- | --- |
| **Title** |  |
| **Journal** |  |
| **Groupings** |  |
| **Mean age** |  |
| **SD** |  |
| **Age range** |  |
| **Gender %Female** |  |
| **Race** |  |
| **N** |  |
| **Locality** |  |
| **Country** |  |
| **Urban or rural ie; socially isolated** |  |
| **Quote: Summarise what the paper is about** |  |
| **Aim: What precisely did it aim to do?** |  |
| **Hypotheses** |  |
| **Recruitment method ie; random (hence representative)** |  |
| **Data collection method** |  |
| **Inclusion/ Exclusion criteria: Check vulnerable group/age etc.** |  |
| **Type of study** |  |
| **Quant/Qual/Grounded in theory** |  |
| **Time: Period of the study and follow up** |  |
| **Response rate** |  |
| **Measures** |  |
| **Pilot Study** |  |
| **Remuneration** |  |
| **Ethical permission** |  |
| **Do participants have history of self harm/suicide attempt/ideation** |  |
| **What are the subgroups of the problem ie; self harm/ideation/previous attempt** |  |
| **Were participants medicated** |  |
| **Did participants have any relation to other self harmer/ family member who had committed suicide?** |  |
| **What was the frequency of internet use?** |  |
| **Follow up studies; What data was missing and why-were these participants different in any way?** |  |
| **What was the internet medium in question ie; website/forum/email/social networking site?** |  |
| **Were the participants using the medium in a positive or negative way (Ie; coping strategy/suicidal methodologies)** |  |
| **Relevant impacts of medium positive/negative/neutral** |  |
| **Did change occur in the participants? If so what? (Ie; coping/thoughts and feelings/support/isolation/ behavioural change (suicide ideation/self-harming behavior)**  **Please include size of effects** |  |
| **What were the mechanisms of change? Did the authors find a mechanism or hypothesize one?** |  |
| **How did participants reach the internet medium in question ie; web site/forum etc.** |  |
| **What were the reasons for use of the medium?** |  |
| **Was the study related to cyber-bullying** |  |
| **Is the internet medium professionally or peer moderated** |  |
| **Is any information missing that the author needs to be contacted for? If so what?** |  |
| **CASP quality score** |  |
| **Authors email** |  |
| **Notes** |  |
