# Supplementary material for: A systematic review of the relationship between internet use, self-harm and suicidal behaviour in young people: The good, the bad and the unknown
Source: PLoS One. 2017 Aug 16;12(8):e0181722. doi: 10.1371/journal.pone.0181722 (PMC5558917; doi:10.1371/journal.pone.0181722)
Supplement: S3 Table — (DOCX) [file pone.0181722.s003.docx]

Supplementary Table 3: Research methodology of reports by CASP quality score

| Casp Score | Quantitative (n=21) | Qualitative (n=18) | Mixed methods (n=12 ) | Total |
| --- | --- | --- | --- | --- |
| High | 13 | 0 | 4 | 17 |
| Medium | 4 | 9 | 6 | 19 |
| Low | 4 | 9 | 2 | 15 |
