# Supplementary material for: Correction: A systematic review of the relationship between internet use, self-harm and suicidal behaviour in young people: The good, the bad and the unknown
Source: PLoS One. 2018 Mar 1;13(3):e0193937. doi: 10.1371/journal.pone.0193937 (PMC5833270; doi:10.1371/journal.pone.0193937)
Supplement: S3 Table — (DOCX) [file pone.0193937.s001.docx]

Supplementary Table 3: Research methodology of reports by CASP quality score

| Casp Score | Quantitative (n=23) | Qualitative (n=16) | Mixed methods (n=12 ) | Total |
| --- | --- | --- | --- | --- |
| High | 14 | 0 | 4 | 18 |
| Medium | 5 | 7 | 6 | 18 |
| Low | 4 | 9 | 2 | 15 |
