# Supplementary material for: Correction: A systematic review of the relationship between internet use, self-harm and suicidal behaviour in young people: The good, the bad and the unknown
Source: PLoS One. 2018 Mar 1;13(3):e0193937. doi: 10.1371/journal.pone.0193937 (PMC5833270; doi:10.1371/journal.pone.0193937)
Supplement: S4 Table — (DOCX) [file pone.0193937.s002.docx]

Supplementary Table 4 Outcomes studied and measures employed in included studies

| **Internet Medium** | **Lead Author, year, country** | **Title** | **Methodology** | **Outcome** | **Outcome measure** |
| --- | --- | --- | --- | --- | --- |
| **General use** | Carew, 2014 [27], Canada and USA | Using digital and social media metrics to develop mental health approaches for youth | Quantitative: digital metrics | Tracking of online behaviours | Software to map online behaviours |
|  | Casiano, 2012 [28],  Canada | Media use and health outcomes in adolescents: Findings from a nationally representative survey | Quantitative: cross sectional survey | Health outcomes  Symptoms of depression and alcohol dependence  Suicide ideation and help seeking | The Canadian Community Health Survey  The Composite International Diagnostic Interview (CIDI) – short form  Questions designed for study |
|  | Carli, 2014 [29],  11 European countries | A newly identified group of adolescents at ‘invisible’ risk for psychopathology and suicidal behaviour: findings from the SEYLE study | Quantitative: cross sectional survey | Risk Behaviours  Psychiatric symptoms  Self-harm | Global School Based Health Survey  Beck Depression Inventory  Zung Self-Rating Anxiety Scale  The Strengths and Difficulties Questionnaire  Paykel Suicide Scale  Deliberate Self-Harm Inventory |
|  | Hagihara, 2012 [26], Japan | Internet suicide searches and the incidence of suicide in young people in Japan | Quantitative: routinely collected data/correlational | Rate of suicide  Monthly figures for suicide-related searches | Statistics from Japanese Ministry of Health, Labour and Welfare  Statistics from Google Insights for Search |
|  | Katsumata, 2008[30], Japan | Electronic media use and suicidal ideation in Japanese adolescents | Quantitative: cross sectional survey | Lifetime history of suicidal ideation  Experience of electronic media usage  Personal communication in everyday life | Self-report questionnaire designed for study |
|  | Kim, 2012 [31], Korea | The nonlinear association between internet using time for non-educational purposes and adolescent health | Quantitative: cross sectional survey | Internet use  Health risk behaviour, mental health (perceived stress, depression, suicidal ideation and attempted suicide) and physical health | Korea Youth Risk Behaviour Web-Based Survey |
|  | Mitchell, 2007 [32], USA | Online behaviour of youth who engage in self-harm provides cues for preventive intervention | Quantitative: cross sectional structured interviews | Online experiences  Internet use characteristics  Self-harm  Psychosocial interactions | The Second Youth Internet Safety Survey (structured telephone interviews)  Youth Self-Report version of the Child Behaviour Check List |
|  | O'Connor, 2014 [33], Northern Ireland | Adolescent self-harm: A school-based study in Northern Ireland | Quantitative: cross sectional survey | Exposure to ‘the troubles’  Excessive internet use  Self-harm  Depression and anxiety  Impulsivity  Self-esteem  Perfectionism | Questionnaire developed for study  The Hospital Anxiety and Depression Scale  Plutchik Impulsivity Scale  Self-Concept Scale  Child and Adolescent Perfectionism Scale |
|  | Robertson, 2012 [3], New Zealand | An adolescent suicide cluster and the possible role of electronic communication technology | Mixed methods: analysis of public health response | Number of suicides  Potential links between cases  Social media use | Data from police inquests, coroners reports and Child Youth Mortality Review database  Interviews with family members  Minutes from meetings between agencies  Accessing relevant publicly available websites |
|  | Collings, 2011 [34], New Zealand | Media influences on suicidal behaviour: an interview study of young people in New Zealand | Mixed methods: cross sectional structured interview | Suicidal behaviours and self-harm  Exposure to and impact of a range of media on suicidal behaviour | Structured interview |
|  | Duggan, 2012 [20], Canada | An examination of the scope and nature of non-suicidal self-injury online activities: Implications for school mental health professionals | Quantitative: cross sectional analysis of search results | Nature and volume of websites returned based on internet searches for self-harm | Counts and categories of search results |
|  | Dunlop, 2011 [35], USA | Where do youth learn about suicides on the internet, and what influence does this have on suicidal ideation? | Quantitative: cross sectional structured interview | Exposure to suicide stories online  Increases in suicide ideation | Interviews |
| **Internet addiction** | Kaess, 2014 [36],  11 European countries | Pathological internet use among European adolescents: psychopathology and self-destructive behaviours | Quantitative: cross sectional survey | Pathological internet use  Depression  Anxiety  Emotional symptoms, conduct problems  Self-harm  Suicide ideation and suicide attempt | Young’s Diagnostic Questionnaire  Beck Depression Inventory  Zung Self-Rating Anxiety Scale  Strengths and Difficulties Questionnaire  Deliberate Self-Harm Inventory  Paykel Suicide Scale |
|  | Kim, 2006 [37], Korea | Internet addiction in Korean adolescents and its relation to depression and suicidal ideation: A questionnaire survey | Quantitative: cross sectional survey | Internet Addiction  Depression  Suicidal ideation | Internet Addiction Scale  Diagnostic Interview Schedule for Children – Major Depression Disorder – Simple  Suicidal Ideation Questionnaire - Junior |
|  | Lam, 2009 [38], China | The association between internet addiction and self-injurious behaviour among adolescents | Quantitative: cross sectional survey | Self-harm  Internet Addiction | Self-report questionnaire  Internet addiction test |
|  | Lin, 2014 [39], Taiwan | The association between suicidality and internet addiction and activities in Taiwanese adolescents | Quantitative: cross sectional survey | Suicide attempt and suicide ideation  Internet addiction and activities  Depression  Self-esteem  Family support | Kiddie Schedule for Affective Disorders  Chen Internet Addiction Scale  Centre for Epidemiological Studies Depression Scale  The Rosenberg Self-Esteem Scale  Family APGAR Index |
|  | Park, 2013 [40], Korea | The association between problematic internet use and depression, suicidal ideation and bipolar disorder symptoms in Korean adolescents | Quantitative: cross sectional survey | Internet addiction  Depression  Suicidal ideation  Diagnosis and symptoms of bipolar disorder | Internet Addiction Proneness Scale for Youth- Short form  Beck Depression Inventory  Reynolds Suicidal Ideation Questionnaire  The Korean Version of the Child Bipolar Questionnaire |
|  | Aktepe, 2013 [41], Turkey | Possible internet addiction in high school students in the city centre of Isparta and associated factors: A cross sectional study | Quantitative: cross sectional survey | Self-harm  Insomnia  Internet addiction  Life satisfaction  Level of loneliness | Survey created for study  Internet Addiction Scale  The Satisfaction with Life Scale  UCLA loneliness Scale |
|  | Messias, 2011 [42], USA | Sadness, suicide, and their association with video game and internet overuse among teens: results from the youth risk behaviour survey 2007 and 2009 | Quantitative: cross sectional survey | Excessive internet use  Sadness  Suicidal ideation  Suicide planning | Youth Risk Behaviour Survey |
| **Online intervention/treatment** | Hetrick, 2014 [43], Australia | Does cognitive behavioural therapy have a role in improving problem solving and coping in adolescents with suicidal ideation? | Quantitative: pre-test/post-test | Problem orientation  Coping  Psychological Distress | The Negative Problem Orientation Questionnaire  Coping Inventory for Stressful Situations  Kessler Psychological Distress Scale |
|  | Hetrick, 2015 [44], Australia | Development and testing of an online monitoring tool of depression symptoms and side effects for young people being treated for depression | Mixed methods: feasibility | Depression  Suicidal ideation  Functionality and usefulness of tool | Patient Health Questionnaire  Suicidal Ideation Questionnaire-Junior  Survey, focus group and interviews |
|  | Mars, 2015 [45], UK | Exploring e-mental health preferences of generation y | Mixed methods: feasibility | Current suicide risk  Psychological symptoms including depression and anxiety  Qualitative data on user views of mental-health information websites | MINI-plus  Brief Depression Inventory  Interview with participants |
|  | Saulsberry, 2013 [46], USA | Randomised control trial of a primary care internet-based intervention to prevent adolescent depression: one-year outcomes | Quantitative: randomised control trial | Depression  Self-harm ideation  Hopelessness  Loneliness  Depression  Support network  Self-efficacy  Motivation  Attitudes  Positive relationships with physician  Adherence to programme  Perceived benefits of programme | Centre for Epidemiological Studies Depression Scale  Patient Health Questionnaire-Adolescent  Perceived Support from Family and Friends/Family  Scales constructed for study |
|  | Barton, 2013 [47], USA | Peer responses to messages of distress: do sex and content matter | Qualitative: content analysis | Type of response to distressed email | Rating of response emails |
|  | Whitlock, 2013 [48], USA | Young adult experiences of disclosing self-injury, suicide-related behaviour, and psychological distress in a web-based survey | Mixed methods: cross sectional survey | Self-harm  Suicidal behaviour  Global psychological distress Co-morbid risk and protective factors  Qualitative data | Non-Suicidal Self-Injury Assessment Tool  Survey of Student Well-Being  Open-ended survey items regarding level of upset and depth of thinking |
| **Social media** | Belfort, 2012 [49], USA | Similarities and differences among adolescences who communicate suicidality to others via electronic versus other means: A pilot study | Quantitative: routine data | Method of communication of distress prior to hospital presentation | Data retrieved from patients notes |
|  | Cash, 2013 [24], USA | Adolescent suicide statements on MySpace | Qualitative: content analysis | Nature of distressed statements on MySpace | Content analysis of statements |
|  | Zdanow, 2012 [21], South Africa | The representation of self injury and suicide on emo social networking groups | Qualitative: content analysis | Portrayal of suicide and self-harm on social networking sites | Content analysis of Facebook groups |
|  | Sueki, 2015 [50], Japan | The association of suicide-related twitter use with suicidal behaviour: a cross-sectional study of young internet users in Japan | Quantitative : cross sectional survey | Twitter use  Suicidal behaviour  Depression  Anxiety | Questionnaire constructed for study |
| **Forums** | Baker, 2008 [51], UK | Understanding self-harm and suicide websites: A qualitative interview study of young adult website users | Qualitative: cohort | Views of users of self-harm/suicide forums | In depth email interviews |
|  | Barak, 2006 [52], Israel | Does activity level in online support groups for distressed adolescents determine emotional relief | Mixed Methods: retrospective cohort | Level of distress | Thematic analysis of messages (Leenaars thematic guide) |
|  | Jones^b^, 2011 [53], UK | Online discussion forums for young people who self-harm: user views | Mixed methods: cohort | Views on discussion forums, forum size and moderation  Themes and illustrative statements | Survey constructed for study  Content analysis of conversation |
|  | McDermott, 2013 [15], UK | Hard-to-reach youth online: methodological advances in self-harm research | Qualitative: content analysis | Examination of forums | Content analysis of forum posts |
|  | Owens^B^, 2012 [54], UK | Building an online community to promote communication and collaborative learning between health professionals and young people who self-harm: an exploratory study | Mixed methods: cohort | Various aspects of forum use | Logs of site activity  Questionnaire constructed for study  Discourse analysis of message board content |
|  | Sharkey^b^, 2012 [55], UK | Supportive interchanges and face-work as ‘protective talk’ in an online self-harm support forum | Qualitative: cohort | Interactions in crisis/support forums | Discourse analysis |
|  | Smithson^b^, 2011 [56], UK | Membership and boundary maintenance on an online self-harm forum | Qualitative: cohort | Relationship formation and maintenance in a self-harm discussion forum | Content analysis |
|  | Smithson^b^, 2011 [57], UK | Problem presentation and responses on an online forum for young people who self-harm | Qualitative: cohort | Problem presentation and responses in an online discussion forum | Content analysis |
|  | Whitlock, 2006 [16], USA | The virtual cutting edge: the internet and adolescent self-injury | Mixed methods: correlational | Content of message board posting  Message board characteristics | Content analysis  Counts of results of internet searches  Data collected from internet service providers |
|  | Eichenberg, 2008 [58], Germany | Internet message boards for suicidal people: a typology of users | Quantitative: cross sectional survey | User characteristics  Usage habits and reasons for use  Effects of the forum | 29 item questionnaire constructed for study |
|  | Franzen, 2011 [17], Sweden | The beauty of blood? Self-injury and ambivalence in an internet community | Qualitative: content analysis | Construction of identity on a self-harm forum | content analysis of forum posts |
|  | McDermott, 2015 [18], UK | Asking for help online: lesbian gay bisexual and trans youth, self-harm and articulating the ‘failed’ self | Qualitative: content analysis | Nature of discussions of suicidal feelings | Thematic analysis of forum posts |
|  | Sueki, 2012 [59], Germany and Japan | Suicide bulletin board systems comparison between Japan and Germany | Quantitative: cross sectional survey | Cross-cultural influence of suicide bulletin board systems | Questionnaire constructed for study |
|  | Westerlund,  2013 [19], Sweden | Talking suicide: online conversations about a taboo subject | Qualitative: content analysis | Nature of conversations about suicide | Content analysis of forum posts |
| **Website with suicide/self-harm content** | Lewis, 2011 [25], Canada | The possible risks of self-injury websites: a content analysis | Qualitative: content analysis | Content of self-harm websites | Content analysis of websites |
|  | Harris, 2013 [60], Cross cultural (UK Europe, Canada, Australia, New Zealand and others) | Exploring the use and effects of deliberate self-harm websites: an internet-based study | Mixed methods: cross sectional survey | Self-harm  Forum use  Reasons for forum use | Questionnaire designed for study |
| **Video/image sharing** | Lewis, 2012 [22], Canada | Helpful or harmful? An examination of viewers responses to non-suicidal self-injury videos of YouTube | Qualitative: content analysis | Nature of comments on self-harm videos | Content analysis of video comments |
|  | Grzanka, 2014 [23], USA | Queer youth suicide and the psycho-politics of ‘it gets better’ | Qualitative: content analysis | Content of YouTube videos | Discourse analysis of videos |
|  | Lewis, 2011 [10], Canada | The scope of non-suicidal self-injury of YouTube | Mixed methods | Quantitative and qualitative variables of YouTube videos | Analysis of videos |
|  | Sternudd, 2012 [61], UK, USA Europe | Photographs of self-injury: production and reception in a group of self-injurers | Mixed methods: cross sectional survey and content analysis | Reasons for looking at self-harm images and reactions to such images | Discourse analysis of forum conversations  Questionnaire constructed for study |
| **Blogs** | Castro^a^, 2012 [62], Portugal and Brazil | Online violence: not beautiful enough, not thin enough. Anorectic testimonials on the web | Qualitative: content analysis | Nature of eating disorder blogs | Content analysis of blogs |
|  | Castro^a^, 2013 [63]^,^ Portugal and Brazil | ‘I love my bones!’ self-harm and dangerous eating youth behaviours in Portuguese written blogs | Qualitative: content analysis | Analysis of blog content to better understand how social and cultural pressures may influence their disruptive behaviours | Content analysis of blogs |
|  | a. Five reports related to the same self-harm forum study (sharptalk)  b. Two reports based on the same set of eating disorder blogs | | | | |
